# Genome-wide single nucleotide polymorphism array analysis unveils the origin of heterozygous androgenetic complete moles

**DOI:** 10.1038/s41598-019-49047-7

**Published:** 2019-08-29

**Authors:** Hirokazu Usui, Kazuhiko Nakabayashi, Kayoko Maehara, Kenichiro Hata, Makio Shozu

**Affiliations:** 1 Department of Reproductive Medicine, Graduate School of Medicine, Chiba University, Chiba, Chiba 260-8670 Japan; 2 Department of Maternal-Fetal Biology, National Research Institute for Child Health and Development, Setagaya, Tokyo 157-8535 Japan; 3 Present Address: Department of Nutrition, Graduate School of Health Sciences, Kio University, Kitakatsuragi, Nara 635-0832 Japan

**Keywords:** Centromeres, Cytogenetics, Centromeres, Uniparental disomy

## Abstract

Hydatidiform moles are abnormal pregnancies, which show trophoblastic hyperplasia. Most often, the nuclear genome in complete hydatidiform moles (CHMs) is composed of only paternal chromosomes. Diploid androgenetic conceptuses can be divided into homozygous and heterozygous CHMs. Heterozygous CHMs originate from two sperms or a diploid sperm, the distinction of which has not been established. Here, we assessed the origin of heterozygous CHMs using single nucleotide polymorphism (SNP) array. Thirteen heterozygous CHMs were analysed using B allele frequency (BAF) plotting to determine the centromeric zygosity status of all chromosomes. One case was from the duplication of a single sperm with an XY chromosome. In the other twelve cases, centromeric zygosity was random, i.e. mixed status. Thus, the twelve heterozygous CHMs were considered to be of dispermic origin but not diploid sperm origin. BAF plotting of SNP array can be a powerful tool to estimate the type of hydatidiform moles.

## Introduction

Hydatidiform moles are abnormal pregnancies characterized by trophoblastic hyperplasia and swelling of the villous structure. They do not result in a baby and are classified into complete hydatidiform mole (CHM) and partial hydatidiform mole (PHM). Approximately 15–20% of CHM and 1–2% of PHM subsequently develop into gestational trophoblastic neoplasia (GTN), which exhibits uterine myometrial lesion and lung metastasis^1,2^. Patients with GTN require chemotherapy, and thus, patients with CHM and PHM should be strictly followed by serum human chorionic gonadotropin (hCG) measurement, a specific and sensitive tumour marker of trophoblastic diseases. Hydatidiform moles are diagnosed by histopathological analysis; however, the accuracy of pathological diagnosis is not enough, especially in PHM^3^, and it is difficult to distinguish between hydatidiform moles and abortions in some cases^3^.

The most CHMs have an exceptional cytogenetic constitution^4^. The nuclear genomes of them are composed of only paternal chromosomes from the sperm (s)^1,4–6^. They are called as androgenetic CHMs, which are diploid and consist of two subtypes—homozygous and heterozygous CHMs. About 90% of androgenetic CHMs are homozygous, in which all the chromosomes are mono-haplotypes, and the rest are heterozygous^5,6^. Heterozygous CHM is sometimes referred to as a dispermic mole, since its chromosome composition does not contradict the dispermic origin^7^. Theoretically, the nuclear genome in a heterozygous CHM could not only be from two sperms, but also from a diploid sperm. The most PHMs are triploid as they contain two paternal chromosomes and one maternal chromosome^8,9^. Further, non-molar villous tissues are usually biparental diploid as they have one paternal and one maternal chromosome, occasionally with aneuploid^1,5,6^. In addition to the histopathological diagnosis of hydatidiform moles, genetic procedures using the nuclear genome obtained from the molar and non-molar villous tissues seem to be useful. The most useful technique is short tandem repeat (STR) polymorphism analysis^1,10,11^, which can discriminate androgenetic homozygous CHM, heterozygous CHM, diandric monogynic triploid, and biparental diploid conceptus. Our group has focused on the molecular genetic diagnosis of molar villous tissues using STR analysis to clarify the actual incidence of GTN from each category^1^. The heterozygous CHM was previously reported to be associated with a higher risk of GTN than the homozygous CHM^12,13^. However, a recent study showed no difference in the incidence of GTN between heterozygous and homozygous CHMs^1^.

The detailed mechanism underlying the development of heterozygous CHM has not been assessed. The PHMs are almost all triploid with diandric monogyny^5,6^. They have a characteristic similar to that of androgenetic heterozygous CHM, i.e. they have two paternal haploids. In some PHMs, the two paternal haploids have been proven to originate from a diploid sperm^2,14^. We hypothesised that heterozygous CHMs with dispermic and diploid origins might show different clinical and biological characteristics.

**Figure 1:**
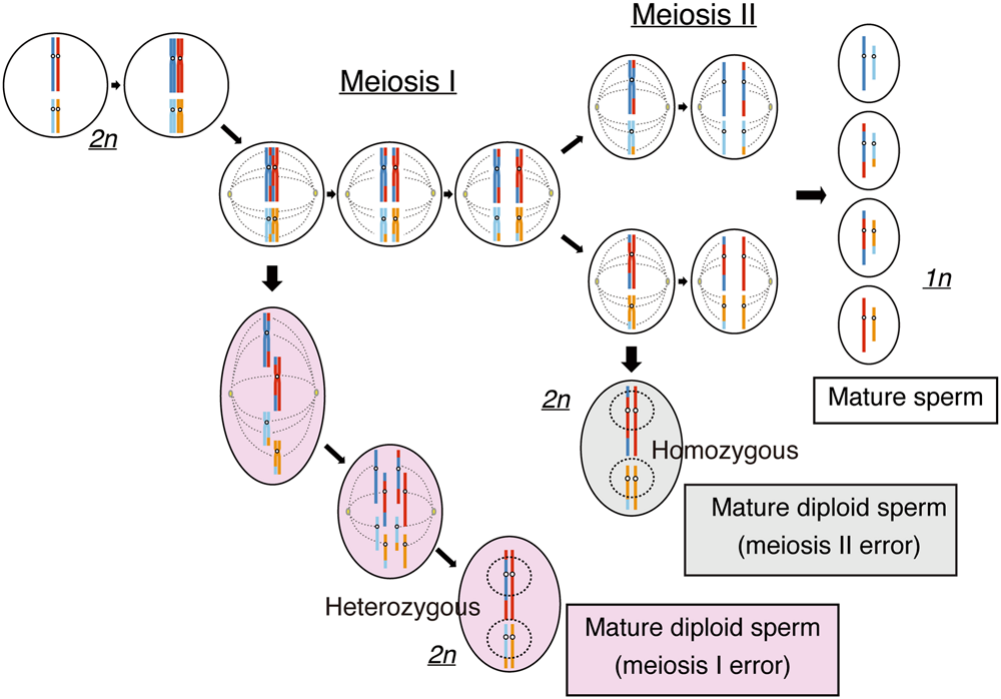
Schematic representation of the development of mature sperm and diploid sperm. The mature sperms were produced after meiosis I and II. Each sperm exhibited a trace of recombination. The mature diploid sperms by meiosis I error were presumed to have all heterozygous centromeric status, whereas the mature diploid sperms by meiosis II error presented all homozygous centromeric status.

A diploid sperm is known to arise by two mechanisms. The first type of diploid sperms is formed by the nonseparation of chromosomes in meiosis I (MI), subsequent meiosis II (MII), and mitosis (Fig. 1). The second type is formed by meiosis II error and subsequent mitosis (Fig. 1). During meiosis for spermatogenesis and oogenesis, recombination of homologous chromosomes occurs. Thus, to determine whether the androgenetic heterozygous CHM originates from a diploid sperm or two independent sperms, it would be useful to discriminate the zygosity status of centromeric regions (hereinafter referred to as the centromeric zygosity), since the zygosities of distal regions from the recombination points should be affected by crossing over. The theoretical centromeric zygosity of heterozygous CHM originating in two sperms should have a random status. However, the centromeric zygosity will be all heterozygous if the CHM originates from a mature diploid sperm with MI error and all homozygous if the CHM originates from a mature diploid sperm with MII error (Fig. 1). The frequency of diploid sperms in the normal population has been estimated to be 0.2–0.3%, whereas that in infertile males is higher (1.1–2.2%)^15^.

Recently, the development of high-density single nucleotide polymorphism (SNP) arrays for genotyping has enabled large-scale SNP studies and comprehensive analysis^16^. Furthermore, the B allele frequency (BAF) algorithm within the Illumina platform can determine the proportion of each allele of the tumour genome^17–19^. BAF plotting of the SNP array can depict and discriminate the homozygous and heterozygous regions of chromosomes, especially in the centromeric region^20^. Therefore, we conducted molecular karyotyping using the SNP array and by BAF plotting of androgenetic heterozygous CHM to elucidate which type of androgenetic heterozygous CHM—dispermic or diploid sperm origin—might be dominant.

## Results

### BAF plots

**Figure 2:**
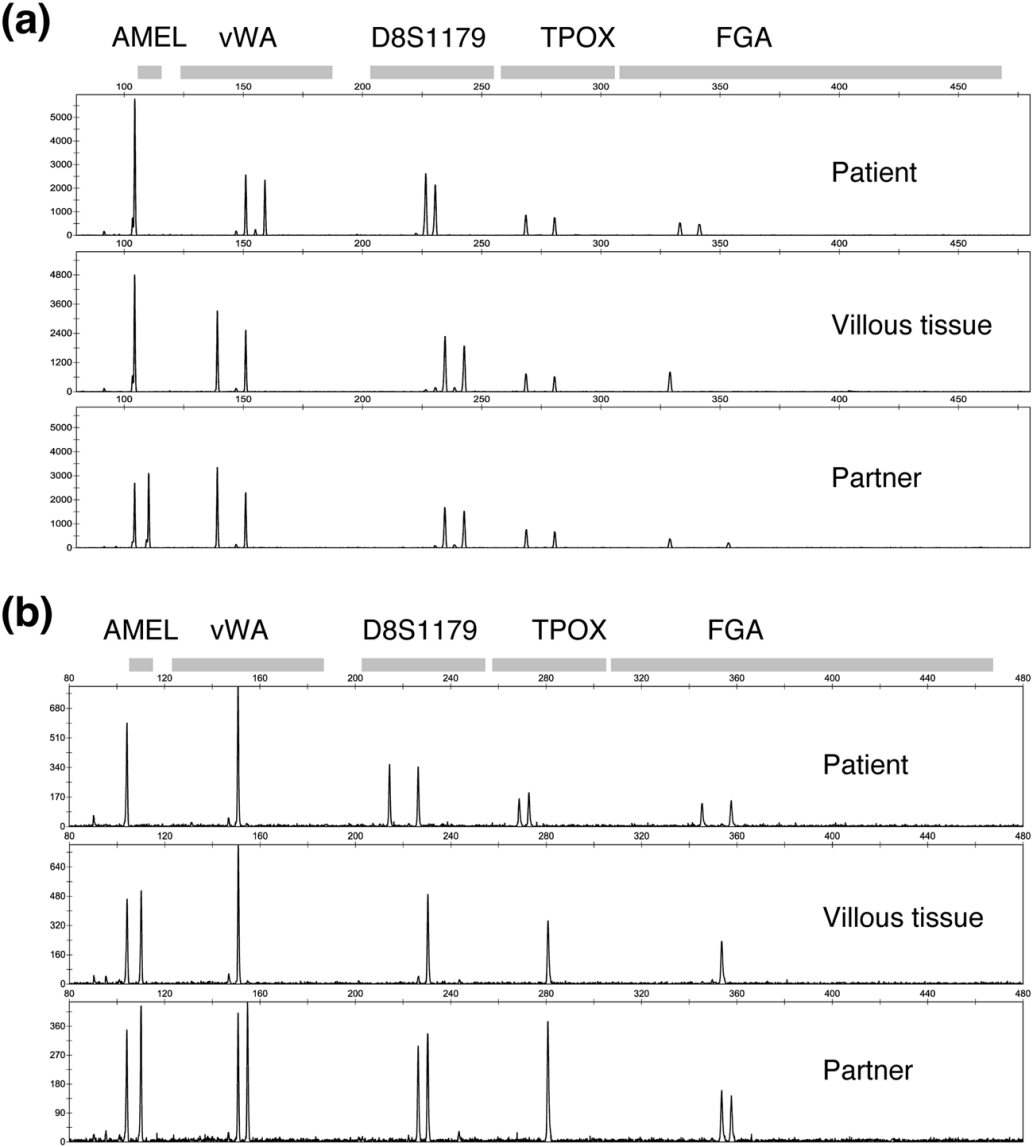
Short tandem repeat polymorphism analysis of heterozygous complete hydatidiform mole. Upper lane, middle lane, and lower lane are the electropherograms of short tandem repeat PCR amplicon obtained from the patient, villous, and partner using the PowerPlex 16HS system. (**a**) HM12; The villous loci of D8S1179 and FGA do not harbour any patient allele. The villous loci of vWA, D8S1179, and TPOX harbour two alleles from partners. (**b**) HM13; The villous amelogenin (AMEL) locus is heterozygous and represents XY pattern.

**Figure 3:**
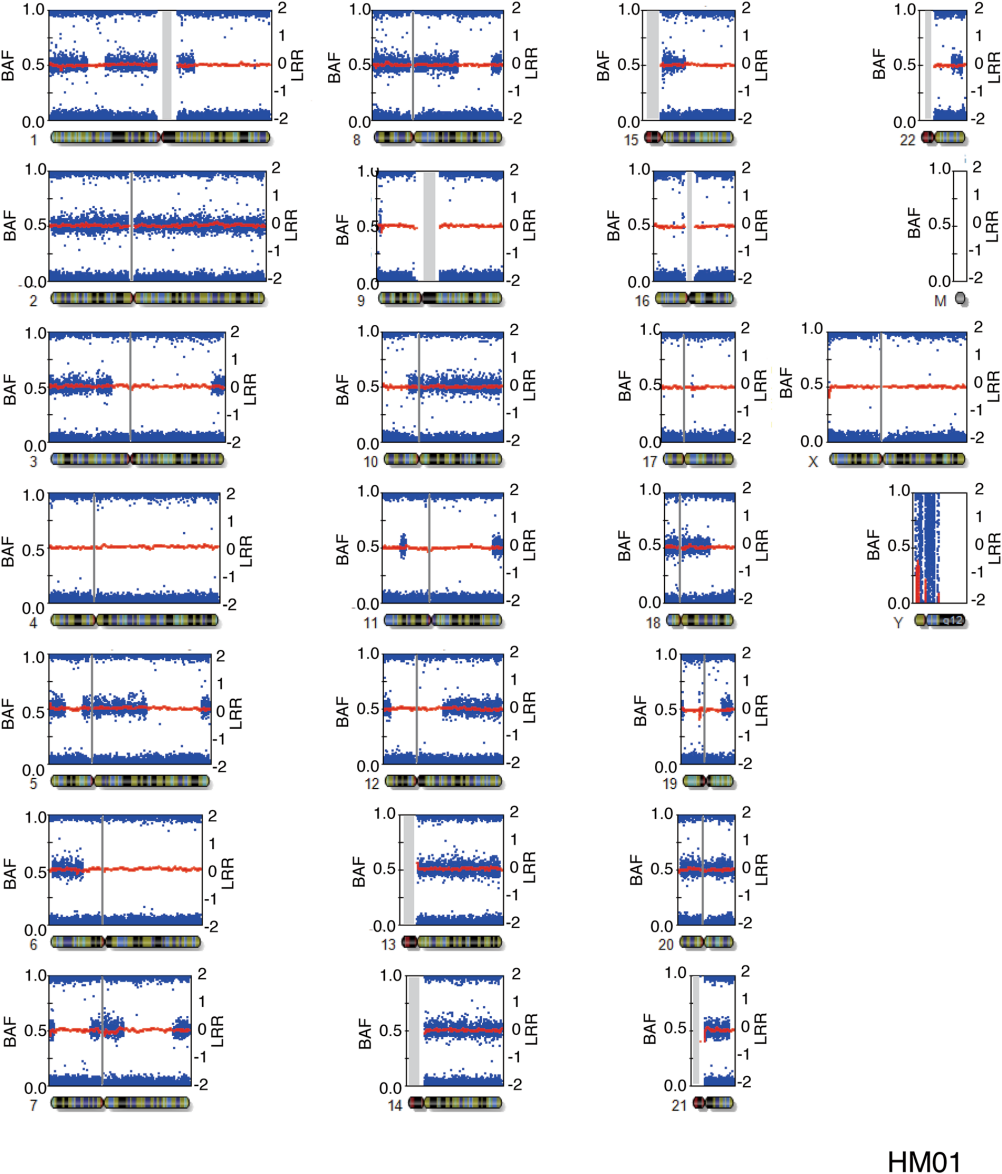
B allele frequency (BAF) and log R ratio plots of the HM01 sample for all chromosomes. No assigned BAF plot was generated for mitochondrial DNA; no effective BAF plot was generated for chromosome Y, since HM01 did not have Y chromosome. Blue dots indicate the BAF values of each probe. Red lines represent the smoothened log R ratio.

**Figure 4:**
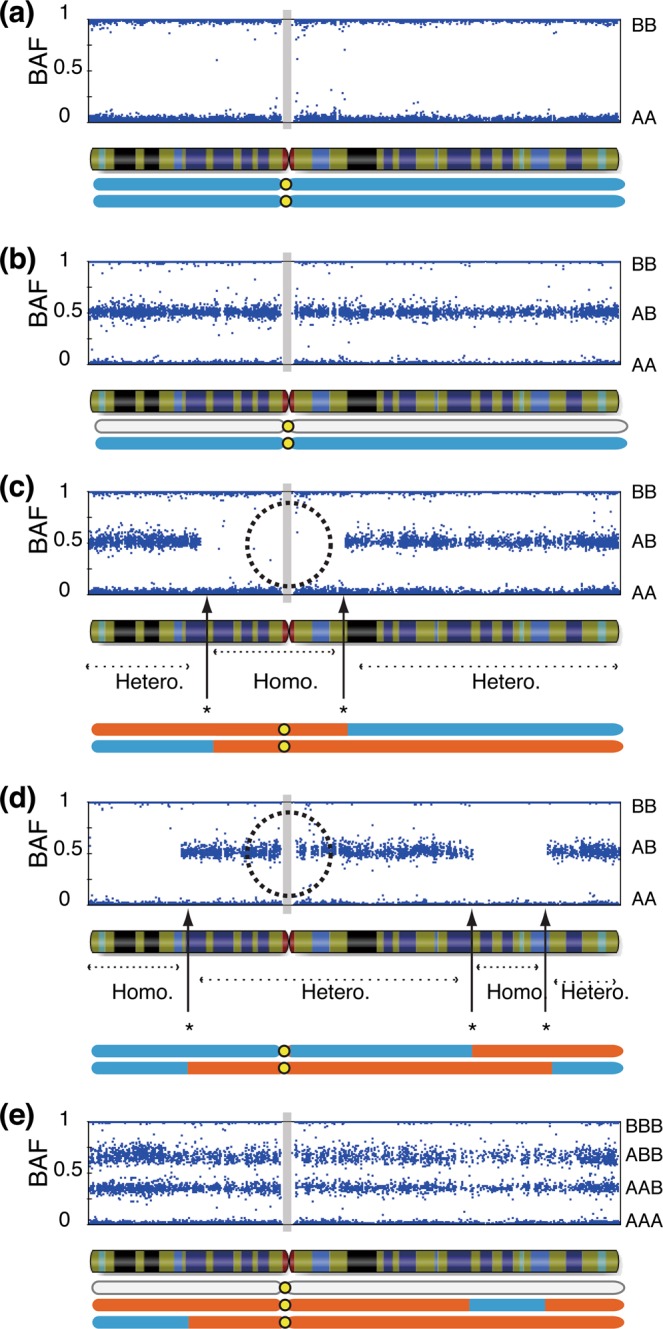
B allele frequency (BAF) plot of villous samples. (**a**) Mono-haploid contribution by single sperm origin. (**b**) BAF plot of biparental diploid case. (**c**) Two haploid paternal contributions with homozygous centromeric status. (**d**) Two haploid paternal contributions with heterozygous centromeric status. (**e**) BAF plot of diandric monogynic triploid case. Grey filled squares are the regions without SNP probes around centromeres. Dotted circles indicate centromeric regions. Orange and cyan regions of the chromosome are of paternal origin. Grey regions of the chromosomes are of maternal origin. Hetero; heterozygous region, Homo; homozygous region. *Recombination point.

Among the patients enrolled in our molecular diagnostic study^1^, we identified 107 androgenetic homozygous CHMs and thirteen androgenetic heterozygous CHMs using STR analysis (Supplementary Table S1), which is the most reliable procedure (Fig. 2)^3,5^. All thirteen samples were pathologically diagnosed as CHM by the certified pathologist. We successfully analysed them using the CytoSNP-12 array. The BAF plots of all the chromosomes were visualised (Fig. 3). If a BAF plotting presented two-lines that consisted of AA and BB, the status of the region was classified as homozygous (Fig. 4a). In normal diploid cells, the BAF plotting presented three-lines—almost 0 as AA, 0.5 as AB, and 1.0 as BB (Fig. 4b). Those regions were defined as heterozygous. In heterozygous CHM diagnosed by STR analysis, both homozygous and heterozygous regions were present, resulting from chromosome recombination (Fig. 4c,d). The breakpoints indicated recombination sites. In triploid or trisomy cases, the BAF plot presented four-lines representing AAA, AAB, ABB, and BBB (Fig. 4e).

The BAF plots of all the samples are shown in Supplementary Fig. S1. In androgenetic heterozygous CHM, the BAF plot of the full chromosome view revealed that the regions showing homozygosity were interspersed throughout the chromosome (Fig. 3 and Supplementary Fig. S1). In HM13, the autosomal chromosomes exhibited all homozygous pattern, although the sex chromosomes indicated an XY pattern (Fig. 2b and Supplementary Fig. S1). Thus, HM13 was re-classified as homozygous androgenetic CHM, which was generated from the endoduplication of mature sperm with XY sex chromosomes (24, XY).

### Zygosity status of centromeric regions

**Figure 5:**
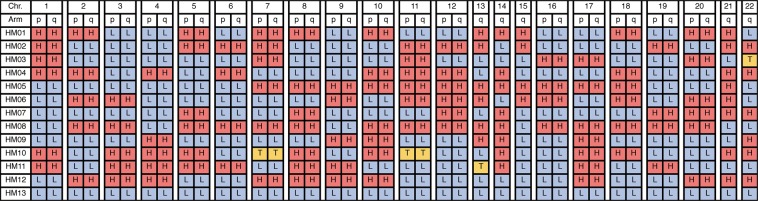
Centromere zygosity of all chromosomes in the analysed samples (HM01-HM13). All B allele frequency plots are presented in Supplementary Fig. S2. Chr., chromosome; p, short arm; q, long arm; H, heterozygosity; L, homozygosity; T, trisomy.

The centromeric zygosity was evaluated under zoom up condition for each chromosome manually on the Illumina GenomeStudio. Figure 4c,d show the centromeric homozygosity and centromeric heterozygosity, respectively. We evaluated the zygosity status of centromeric regions of all the autosomal chromosomes. The centromeric regions of both short and long arms of metacentric and sub-metacentric chromosomes were evaluated. The centromeric zygosities of both short and long arm sides were separately recorded. Only the status of long arms was evaluated in the centromeric region of acrocentric chromosomes. In total, 264 centromeric statuses of 22 chromosomes in 12 cases (except HM13) were evaluated. We could not classify four centromeric regions because those chromosomes were trisomic (Supplementary Fig. S1; chromosome 22 in HM03, chromosome 7 in HM10, chromosome 11 in HM10, and chromosome 13 in HM11). However, we could classify the status of the other 260 centromeric sites. The summarised zygosity status of centromeric regions is shown in Fig. 5. We identified heterozygosity in 127 sites and homozygosity in 133 sites (Table 1).

**Table 1:** Estimated molecular karyotyping results and summary of centromeric zygosities.

Sample	Estimated karyotype	X	Y	No. of centromeric heterozygosities	No. of centromeric homozygosities
HM01	46,XX	XX	—	12	10
HM02	46,XX	XX	—	11	11
HM03	47,XY,+22	X	Y	6	15
HM04	46,XY	X	Y	11	11
HM05	47,XYY	X	YY	13	9
HM06	46,XY	X	Y	11	11
HM07	46,XX	XX	—	8	14
HM08	46,XY	X	Y	16	6
HM09	46,XX	XX	—	7	15
HM10	48,XX,+7,+11	XX	—	11	9
HM11	47,XY,+13	X	Y	10	11
HM12	46,XX	XX	—	11	11
Total				127	133
HM13	48,XXYY	XX	YY	0	22

### Classification of heterozygous CHM origin

One heterozygous androgenetic CHM (HM13) was re-classified as homozygous androgenetic CHM, namely the monospermic CHM with sex chromosome tetrasomy because the BAF plots were concordant with the endoduplication of mature sperm with XY sex chromosomes (24, XY). All the remaining twelve cases presented homozygous and heterozygous statuses in the centromeric region except trisomic chromosomes. The number of heterozygotic and homozygotic centromeric regions ranged from 6 to 16 and 6 to 15, respectively (Fig. 5 and Table 1). If androgenetic heterozygous CHMs were to arise from a diploid sperm, the centromeric status would be all homozygous or all heterozygous without exception. Thus, we concluded that the twelve androgenetic heterozygous CHMs in the present study originated from two mature sperms, namely two random haploid sperms, but not from a diploid sperm.

### Molecular karyotyping with BAF and LRR

**Figure 6:**
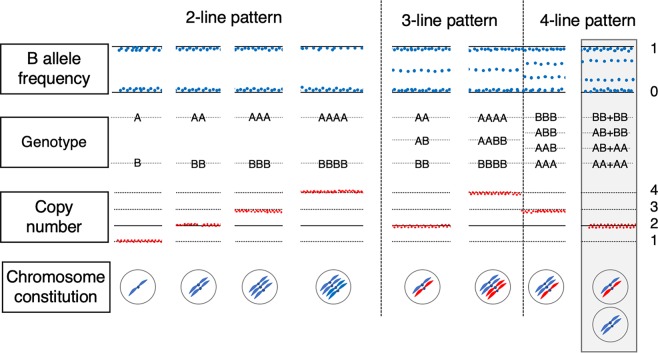
Relationship between BAF plotting, estimated genotypes, and the theoretical copy number and chromosomal constitution. The presumed combinations of B allele frequency, genotypes, copy number, and chromosomal constitution are presented. The grey shading part is the theoretical BAF plot model of mosaic tissue with two cell lines, including biparental diploid and androgenetic diploid cells.

We estimated the karyotypes of 13 villous tissue samples. Molecular karyotyping was performed using BAF plots and log R ratio (LRR) data. The algorithms for the estimation of the genotypes based on the copy number and BAF plot patterns are depicted in Fig. 6. The four-line patterns of the BAF plot indicate that the genotypes were AAA, AAB, ABB, and BBB, and the copy number was three in a case with homogenous cell population. Contrastingly, the two- and three-line patterns of the BAF plots could not determine the genotypes because the different copy numbers would result in different genotypes (Fig. 6). In rare frequency, the theoretical BAF plot would present four-lines in mosaic cases with one diploid androgenetic cell line and one diploid cell line with the biparental nuclear genome (Fig. 6)^21^.

**Figure 7:**
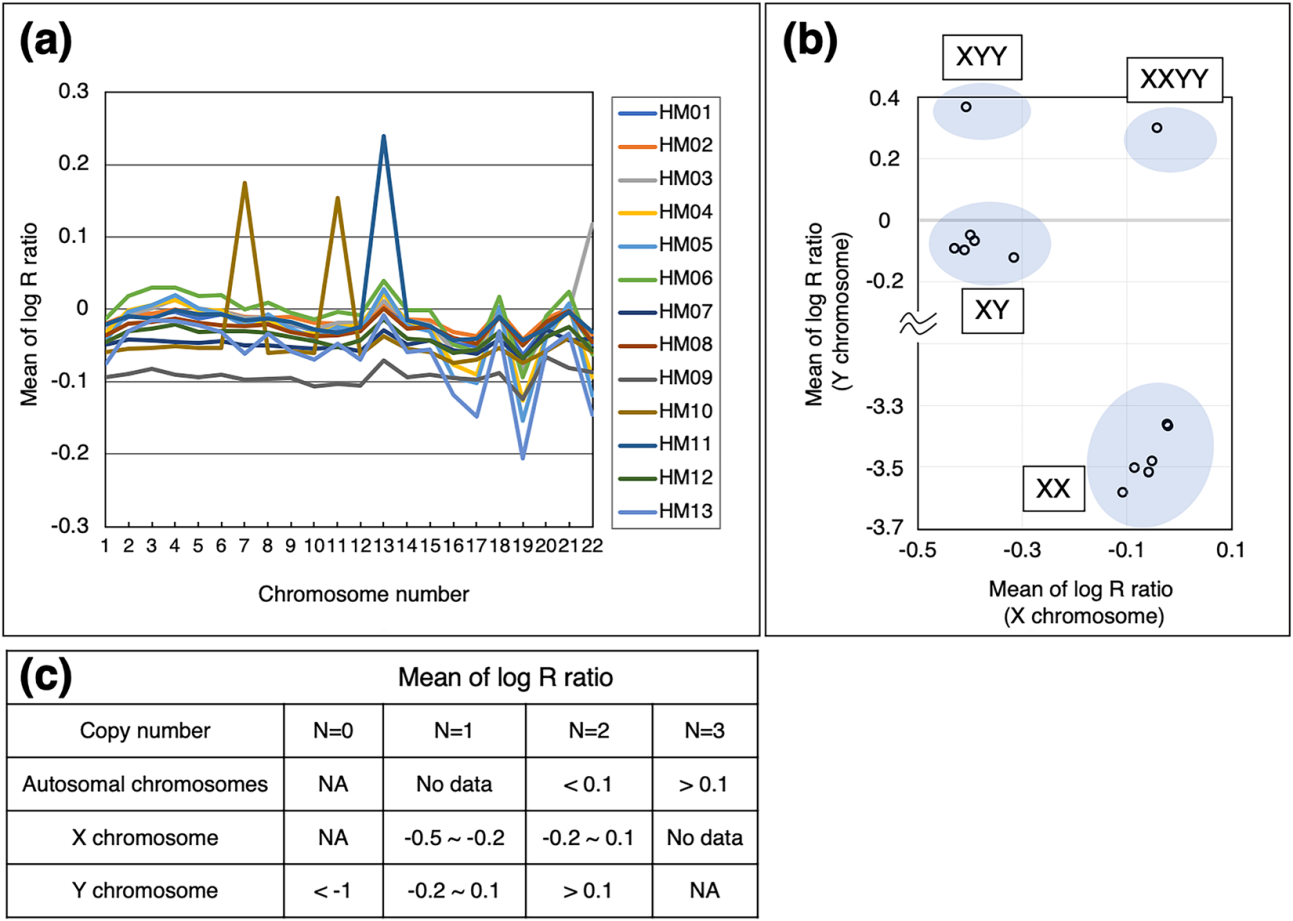
Distribution of means of log R ratio per chromosome and algorithm for the estimation of copy number from log R ratio. (**a**) Means of log R ratio on autosomal chromosomes are plotted. (**b**) Scatter plot of the mean of log R ratio between X and Y chromosomes. Blue shading ellipses are indicated as the estimated groups of sex chromosome types. (**c**) Estimated ranges of log R ratio per chromosome (autosomal, X, and Y chromosomes) are depicted. NA, not applicable.

LRR is the logged ratio of observed probe intensity to expected intensity. Thus, any deviations from zero in this metric are evidence for copy number change^19^. To determine the copy number per chromosome based on the LRR, we calculated the mean and standard deviation of the LRR for each chromosome (Supplementary Table S2). The plots of the mean LRR for chromosomes in every sample are depicted in Fig. 7a. Four autosomal chromosomes (chromosome 22 in HM03, chromosome 7 in HM10, chromosome 11 in HM10, and chromosome 13 in HM11) showed trisomy, as their BAF plots showed the four-lines—AAA, AAB, ABB, and BBB (Supplementary Fig. S1). The mean LRR for the four trisomic chromosomes was >0.1 (Fig. 7a). Further, the mean of LRR ranged from −0.1 to 0.1, indicating that almost all the chromosomes were disomic (Fig. 7a, and Supplementary Figs. S2). We compared the distribution of LRR between the trisomic and disomic chromosomes. The distribution of the LRR in trisomic chromosomes marginally shifted to the right (over zero) (Supplementary Fig. S3). Aneuploidy could be determined using only LRR information. However, the mean of LRR showed a little variation. Based on the data of raw LRR plotting and standard deviation of LRR, the larger standard deviation of LRR induced unstable distribution of LRR as HM09 (Fig. 7a and Supplementary Table S2).

### Sex chromosomes constitutions

The interpretation of the BAF plots and LRR data for sex chromosomes is slightly complicated. When the mean value of LRR is around zero, the X chromosome is speculated to be disomic as in autosomal chromosomes. However, in the case of the Y chromosome, an LRR close to zero is indicative of a single copy. The means of LRR for X and Y chromosomes, obtained from all thirteen samples, are plotted in Fig. 7b. The plots were grouped into four categories, namely XX, XY, XXYY, and XYY, as shown in Fig. 7b. The plots distributed in the upper-left corner of Fig. 7b contained two groups having one X chromosome. On the other hand, the plots distributed in the right half of the figure contained two groups with two X chromosomes. The estimated ranges of the LRR corresponding to the copy number are shown in Fig. 7c. The histograms of the distribution of LRR on sex chromosomes could further determine the sex chromosome constitution (Supplementary Fig. S4). The left shifts of the peaks indicated one X chromosome. In the case of the Y chromosome, no peaks indicated the absence of the Y chromosome, and the right shifts of the peaks indicated two Y chromosomes. We have summarised the estimated karyotypes of all the samples in Table 1.

In presence of the Y chromosome, the BAF plotting of the X chromosome showed an interesting presentation (Supplementary Fig. S5a,e,f). The end of the short arms on the X and Y chromosomes of HM03 indicated a disomic pattern, although the case of HM03 should have one X chromosome (Supplementary Fig. S5a). Considering the BAF plots of sex chromosomes, two pseudoautosomal regions (PARs) and other homologous genes in sex chromosomes should be considered^22,23^. The X and Y chromosomes are genetically distinct and not completely homologous. However, a half of Y chromosome genes are homologs on the X chromosome^22^. PAR1 and PAR2 are located at the terminal of the short and long arms of the X and Y chromosomes, which share homologous sequences (Supplementary Fig. S5a)^23^. The CytoSNP-12 array includes “XY” probes in addition to “X” and “Y” probes. The allocations of “XY” probes are assigned in PAR1, PAR2, and the centromeric region of only the short arm of the X chromosome but not that of the Y chromosome (Supplementary Fig. S5c). Some SNP loci, which shared sequences between the X and Y chromosomes were not assigned to ‘XY’ probes (Supplementary Fig. S5b, black dotted circle). The existence of counterpart homologous sex chromosomes could explain the BAF plots observed such as those for disomic X and disomic Y (from different males’ origin) in the XY case (Supplementary Fig. S5a). We classified the sex chromosomes of HM05 as XYY based on the histogram of LRR data and mean of LRR (Supplementary Figs S4 and 7b). The BAF plots for the trisomic region of PAR1 of HM05 could also be similarly understood. In total, three counterparts (one X and two Y) could explain the trisomic pattern (AAA, AAB, ABB, and BBB) (Supplementary Fig. S5e). For the tetrasomy (XXYY) of HM13, the disomic BAF phenomenon of PAR1 could be explained by the complete duplication of XY (AAAA, AABB, and BBBB) (Supplementary Fig. S5f). Finally, we estimated the six aneuploids as autosomal trisomy (47,XY,+22, 47,XY,+13, and 48,XX,+7,+11), sex chromosome trisomy (47,XYY), and sex chromosome (48,XXYY).

## Discussion

We used BAF plotting to reveal the centromeric zygosity status in androgenetic heterozygous CHMs and to clarify that the heterozygous CHM is of dispermic origin. The meanings of the terms dispermic CHMs and heterozygous CHMs are the same as previously described^7,24^. To our knowledge, no study has investigated whether the heterozygous CHM is of dispermic or abnormal diploid sperm origin. BAF plotting can overcome the two limitations of centromeric STR analysis: the small loci number and the distance between the centromere and STR loci. The number of markers was enough to evaluate all chromosomes, and the nearest makers were sufficiently close to the centromere as reported in our recent study^20^. In the present study, we proved that the centromeric statuses were randomly homozygous and heterozygous. Cases with heterozygous CHM were determined to be of dispermic origin.

We performed molecular karyotyping using the BAF and LRR. The most striking result in the BAF plot of the heterozygous androgenetic CHM was the copy number neutral homozygous regions. This phenomenon is due to spermatogenic meiosis, during which recombination occurs. Two sperm fertilisation will result in segmental copy number neutral homozygous regions. Another example of segmental copy number neutral homozygous regions is ovarian teratoma^20^. Ovarian teratomas exhibit a pattern similar to that of segmental copy number neutral homozygous regions throughout the genome, although the centromeric status is different from the dispermic CHM^20^. Further, they exhibit all homozygous or all heterozygous centromeric status, but not a mixed status and develop from oocytes with a meiotic error. This is contrary to heterozygous CHMs, which show a mixed status of centromeres.

To the best of our knowledge, the present study is the largest and the first to report the uniparental disomic pattern of more than ten heterozygous CHMs in the entire genome. Some studies have used the SNP array to analyse hydatidiform mole^9,25–31^. CHMs were used as a mono-haploid genomic source material^25–28^. The SNP array was used as a tool for villous classification^9,30,31^. Bug *et al*. mentioned the diagnostic utility of uniparental isodisomy for CHM in one step analysis similar to this study^30^.

Four cases among twelve heterozygous dispermic androgenetic CHMs had aneuploid chromosomes. The parental origin of aneuploid chromosomes was not unveiled in the present study. However, one-third of the heterozygous CHM cases had additional chromosomes. Two studies have reported androgenetic CHM with trisomy of chromosome 11^11,32^. Both studies proved that the additional chromosome should be of maternal origin. Interestingly, all reported cases with a trisomy were androgenetic heterozygous CHMs but not homozygous CHMs^11,32–34^. In our series of more than one hundred homozygous androgenetic CHMs, we did not observe trisomy cases with maternal contribution^1^. The presence of trisomy might be related to the specific developmental mechanism of dispermic CHMs.

There are a few limitations of the present study. First, the number of analysed cases was small. One of the reasons for this is the rarity of heterozygous androgenetic CHMs. The heterozygous CHM is observed in about 10–15% of androgenetic CHMs^1,5,6^. In addition, the STR analysis for villous tissues is not a general examination and is expensive. More definitive results require a higher number of cases with heterozygous CHMs, which necessitates a nationwide study. Second, the molecular karyotyping using SNP array have the methodological limitation. LRR is calculated from the deviations from zero, which would be determined based on the signal intensity of the dominant ploidy. Thus, aneuploidy could be determined, but polyploidy like triploidy or tetraploidy could not be classified only with LRR. Cytogenetic procedures as conventional karyotyping or fluorescence *in situ* hybridization could help the exact determination of ploidy, although we had not performed them.

In conclusion, BAF and LRR plotting of SNP array can be a powerful tool to estimate the type of hydatidiform moles. The high-density SNP array data revealed that the heterozygous CHMs were of dispermic origin. Molecular karyotyping results of the present study revealed a marginally high rate of trisomy in androgenetic heterozygous CHMs.

## Methods

### Ethics approval and consent to participate

The studies were approved by the Biomedical Research Ethics Committee of the Graduate School of Medicine, Chiba University (Approval reference No. 673 and 884). Written informed consents were obtained from all patients before participation, in accordance with the Declaration of Helsinki.

### Sample collection

Between 2007 and 2012, 197 patients enrolled in the molecular diagnosis study on molar pregnancy (Approval reference No. 673). The villous tissues and blood samples were collected from the patients. Partners of some patients also joined the study^25^.

### DNA preparation and STR polymorphism analysis

The genomic DNA of the villous tissue and blood was extracted using the QIAamp^®^ DNA Mini Kit (Qiagen, Hilden, Germany) according to the instruction of the manufacturer. The genomic DNA concentration was quantified by measuring the absorbance at 260 nm using a NanoDrop 1000 spectrometer (Thermo Fisher Scientific Inc., Waltham, MA). The short tandem repeat (STR) polymorphism analysis was performed using the PowerPlex^®^ 16 or PowerPlex^®^ 16 HS System (Promega, Madison, WI), as previously described^1,12^. The resulting amplicons were analysed using the ABI Prism 310 Genetic Analyzer (Applied Biosystems, Inc., Foster City, CA) and GeneMapper software version 4.0 (Applied Biosystems, Inc.). The genetic diagnosis of molar pregnancy was carried out as described previously^1,12^. If one or more villous loci did not present any maternal alleles, they were classified as androgenetic. If the androgenic CHM had at least one locus with two different alleles of paternal origin, it was considered heterozygous CHM (Fig. 2). The androgenic CHM with only one allele of paternal origin in all the loci was classified as homozygous CHM.

Thirteen patients (HM01–HM13) with androgenetic heterozygous CHM diagnosed by the STR analysis were recruited for the present study (Approval reference No. 884).

### SNP array analysis and BAFs

The genomic DNA from the villous tissues of thirteen androgenetic heterozygous CHMs were analysed by the Illumina Human CytoSNP-12 SNP v2.1 BeadChip arrays, containing 292,518 probes, covering the entire genome according to the protocol of the manufacturer (Illumina, Inc., San Diego, CA)^20^. Briefly, 200 ng of genomic DNA was used for array analysis. DNA amplification, tagging, and hybridisation were performed on iScan (Illumina, Inc.). The raw data were normalised in GenomeStudio v2011.1 (Illumina, Inc.) using the information contained within the array. We obtained the BAF and log R ratio (LRR) using the Genotyping module (v1.9.4) in GenomeStudio v2011.1 (Supplementary Figs S1 and S2)^17,19^. For determining the sex chromosome constitution, we used the filtered function with “XY probe” (default setting), without “XY probe”, and only with “XY probe” using GenomeStudio software. The SNP array data are accessible through the Gene Expression Omnibus (GEO) database under accession number GSE 117672 (NCBI, http://www.ncbi.nlm.nih.gov/).

### Zygosity of the centromeric region

The zygosity of the centromeric region was evaluated by generating the BAF plots for both sides of the centromere if the chromosomes were metacentric or sub-metacentric or only for the long arm if the chromosomes were acrocentric (Fig. 4c,d).

### Determination of the developmental mechanism of heterozygous CHM

Based on the developmental mechanism, cases with (1) diploid sperms that originated with MI error were defined as those who showed heterozygosity at all centromeres and (2) diploid sperms that originated with MII error were defined as those who showed homozygosity at all centromeres (Fig. 1)^35^. If the centromere status was mixed, the case was considered to be of dispermic origin.

### Statistical analysis

Statistical analyses were performed using R software v.3.5 (http://www.r-project.org/) and Microsoft Excel.

## Supplementary information

Supplementary information

Supplementary TableS2

## Data Availability

The datasets generated during and analysed during the current study are available from the corresponding author on reasonable request.
