# Supplementary material for: Genome-wide single nucleotide polymorphism array analysis unveils the origin of heterozygous androgenetic complete moles
Source: Sci Rep. 2019 Aug 29;9:12542. doi: 10.1038/s41598-019-49047-7 (PMC6715694; doi:10.1038/s41598-019-49047-7)
Supplement: Supplementary file 1 — Supplementary information [file 41598_2019_49047_MOESM1_ESM.pdf]

Supplementary information

Genome-wide single nucleotide polymorphism array analysis unveils the origin of heterozygous androgenetic complete moles

Hirokazu Usui[1], Kazuhiko Nakabayashi[2], Kayoko Maehara[2,3], Kenichiro Hata[2], Makio Shozu[1]

[1]Department of Reproductive Medicine, Graduate School of Medicine, Chiba University, Chiba, Chiba 260-8670, Japan

[2]Department of Maternal-Fetal Biology, National Research Institute for Child Health and Development, Setagaya, Tokyo 157-8535, Japan

[3]Present address: Department of Nutrition, Graduate School of Health Sciences, Kio University, Kitakatsuragi, Nara 635-0832, Japan

Correspondence and requests for materials should be addressed to H.U. (email: hirokazu-usui@facultychiba-u.jp)

Supplementary Information

Supplementary Table S1: Results of molecular diagnosis performed by short tandem repeat polymorphism analysis of molar pregnancy

Supplementary Figure S1: B allele frequency plotting of all heterozygous androgenetic complete hydatidiform moles

Supplementary Figure S2: Log R ratio plotting of all heterozygous androgenetic complete hydatidiform moles

Supplementary Figure S3: Distribution of log R ratio of chromosomes 7, 11, 13, and 22

Supplementary Figure S4: Histograms of distribution of log R ratio on sex chromosomes

Supplementary Figure S5: B allele frequency (BAF) plots and log R ratio (LRR) of sex chromosomes

Supplementary Table S1

Results of molecular diagnosis performed by short tandem repeat polymorphism analysis of suspected molar pregnancy between 2007 and 2012

Genetic classification		(*n*)	Estimated pathology
Androgenetic diploid	homozygous	107	CHM
	heterozygous	13	
Diandric monogynic triploid		25	PHM
Biparental diploid		35	Non-molar
Monoandric digynic triploid		1	
Failure to analyse		16	
Total		197	

CHM, complete hydatidiform mole; PHM, partial hydatidiform mole

Supplementary Figure S1:

B allele frequency plotting of all heterozygous androgenetic complete hydatidiform moles. Grey lines and squares indicate the regions without SNP probes.

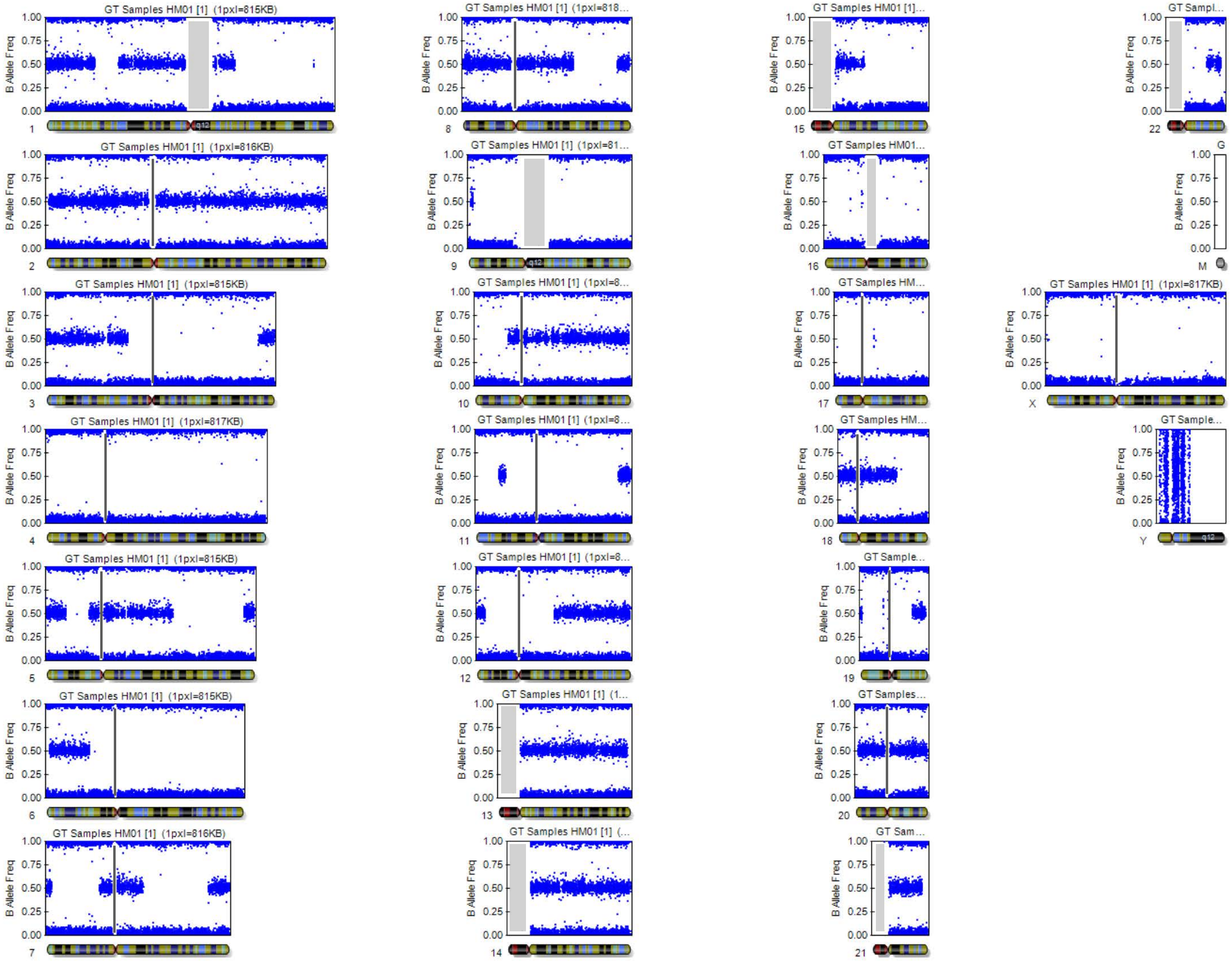
GT Samples HM01 [1] (1pxl=815KB)
GT Samples HM01 [1] (1pxl=816KB)
GT Samples HM01 [1] (1pxl=817KB)
B Allele Freq
1.00
0.75
0.50
0.25
0.00
1
2
3
4
5
6
7
8
9
10
11
12
13
14
15
16
17
18
19
20
21
22
X
Y
M
G
q12

HM01

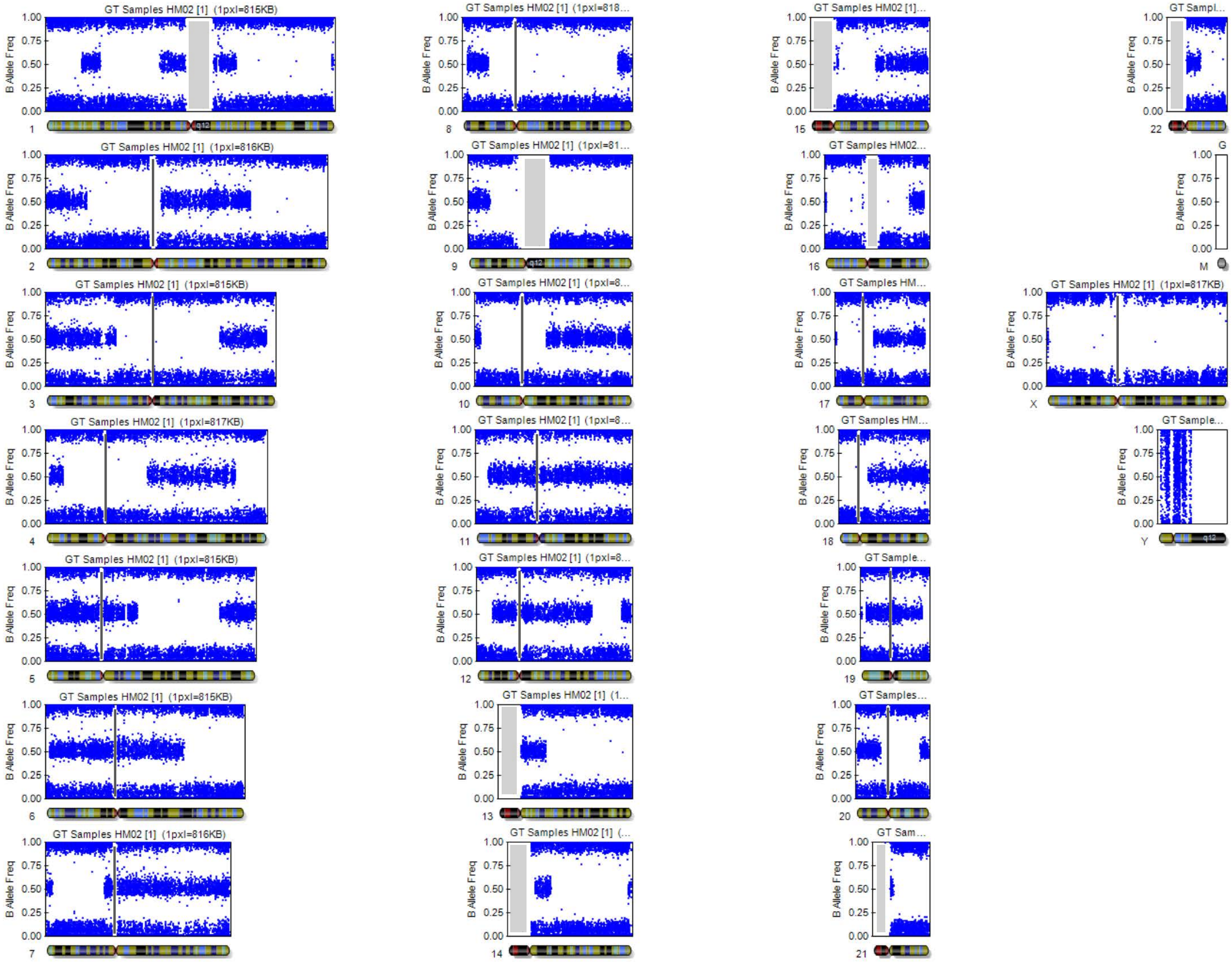
GT Samples HM02 [1] (1pxl=815KB)
GT Samples HM02 [1] (1pxl=816KB)
GT Samples HM02 [1] (1pxl=815KB)
GT Samples HM02 [1] (1pxl=817KB)
GT Samples HM02 [1] (1pxl=815KB)
GT Samples HM02 [1] (1pxl=815KB)
GT Samples HM02 [1] (1pxl=816KB)
GT Samples HM02 [1] (1pxl=817KB)
B Allele Freq
1.00
0.75
0.50
0.25
0.00
1
2
3
4
5
6
7
8
9
10
11
12
13
14
15
16
17
18
19
20
21
22
X
Y
M
G
q12

HM02

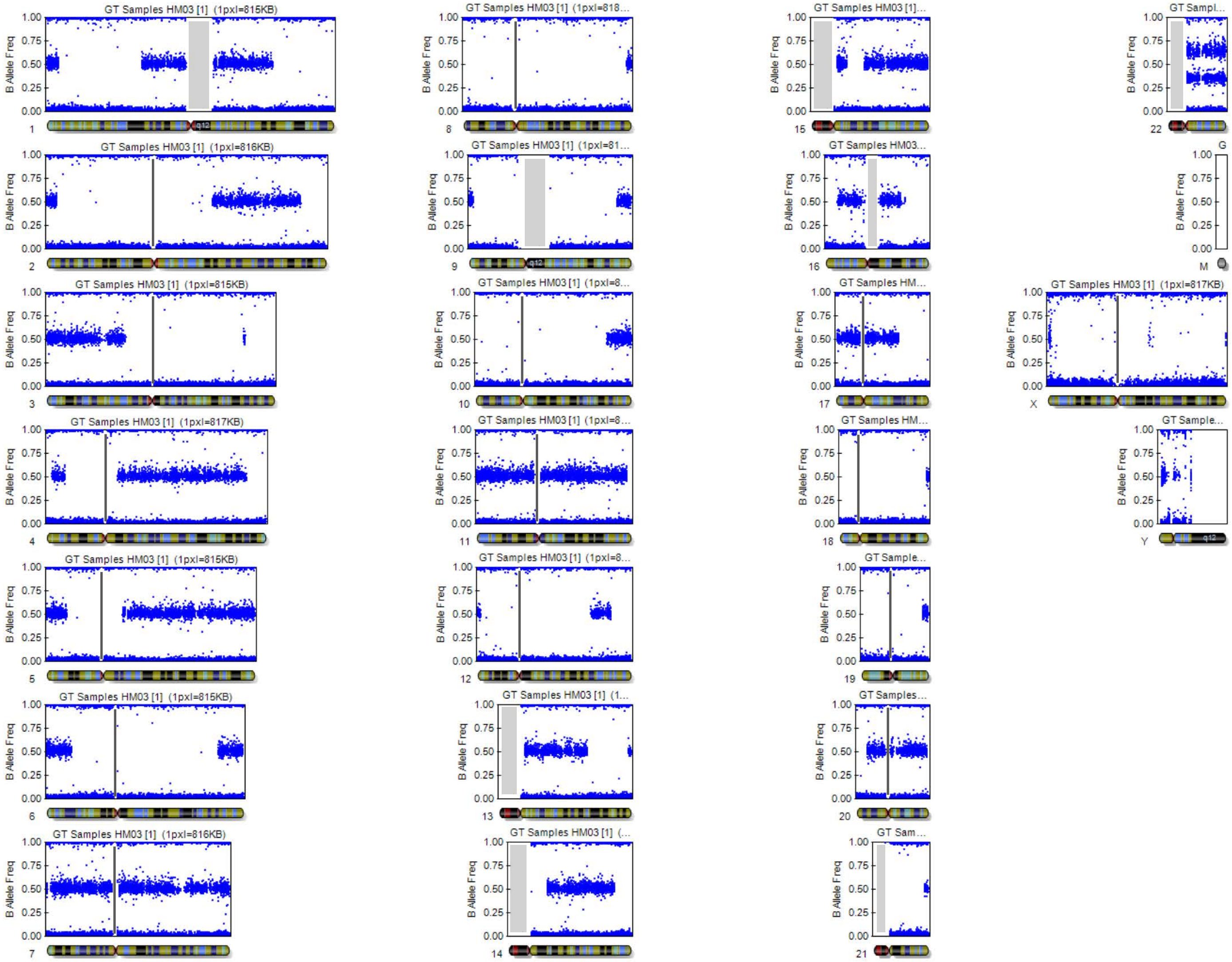
GT Samples HM03 [1] (1pxl=815KB)
GT Samples HM03 [1] (1pxl=816KB)
GT Samples HM03 [1] (1pxl=817KB)
B Allele Freq
1.00
0.75
0.50
0.25
0.00
1
2
3
4
5
6
7
8
9
10
11
12
13
14
15
16
17
18
19
20
21
22
X
Y
M
G
q12

HM03

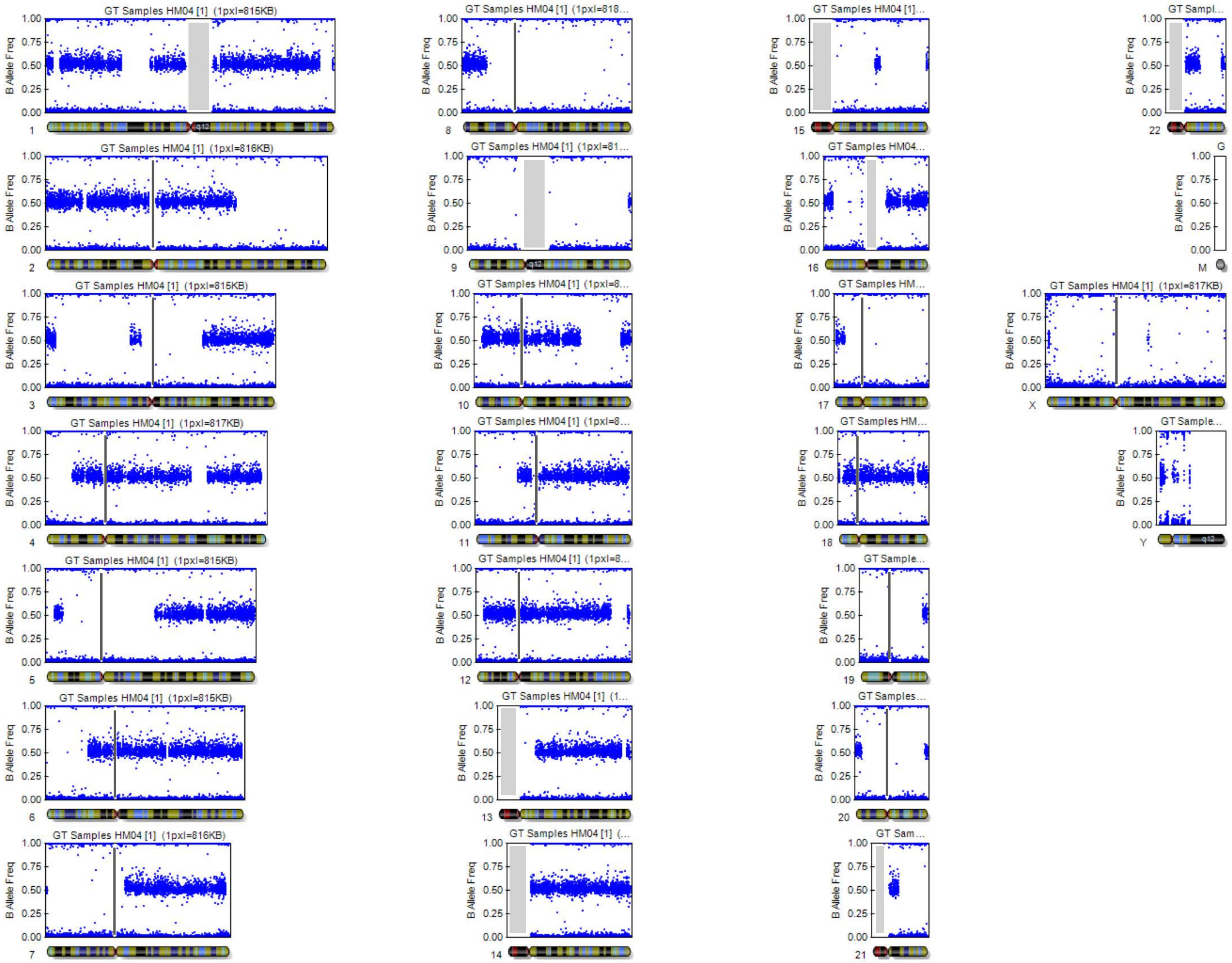
GT Samples HM04 [1] (1pxl=815KB)
GT Samples HM04 [1] (1pxl=816KB)
GT Samples HM04 [1] (1pxl=815KB)
GT Samples HM04 [1] (1pxl=817KB)
GT Samples HM04 [1] (1pxl=815KB)
GT Samples HM04 [1] (1pxl=815KB)
GT Samples HM04 [1] (1pxl=816KB)
GT Samples HM04 [1] (1pxl=818...
GT Samples HM04 [1] (1pxl=81...
GT Samples HM04 [1] (1pxl=8...
GT Samples HM04 [1] (1pxl=8...
GT Samples HM04 [1] (1pxl=8...
GT Samples HM04 [1] (1...
GT Samples HM04 [1] (...
GT Samples HM04 [1]...
GT Samples HM04...
GT Samples HM...
GT Samples HM...
GT Sample...
GT Samples...
GT Sam...
GT Sampl...
GT Samples HM04 [1] (1pxl=817KB)
GT Sample...
B Allele Freq

HM04

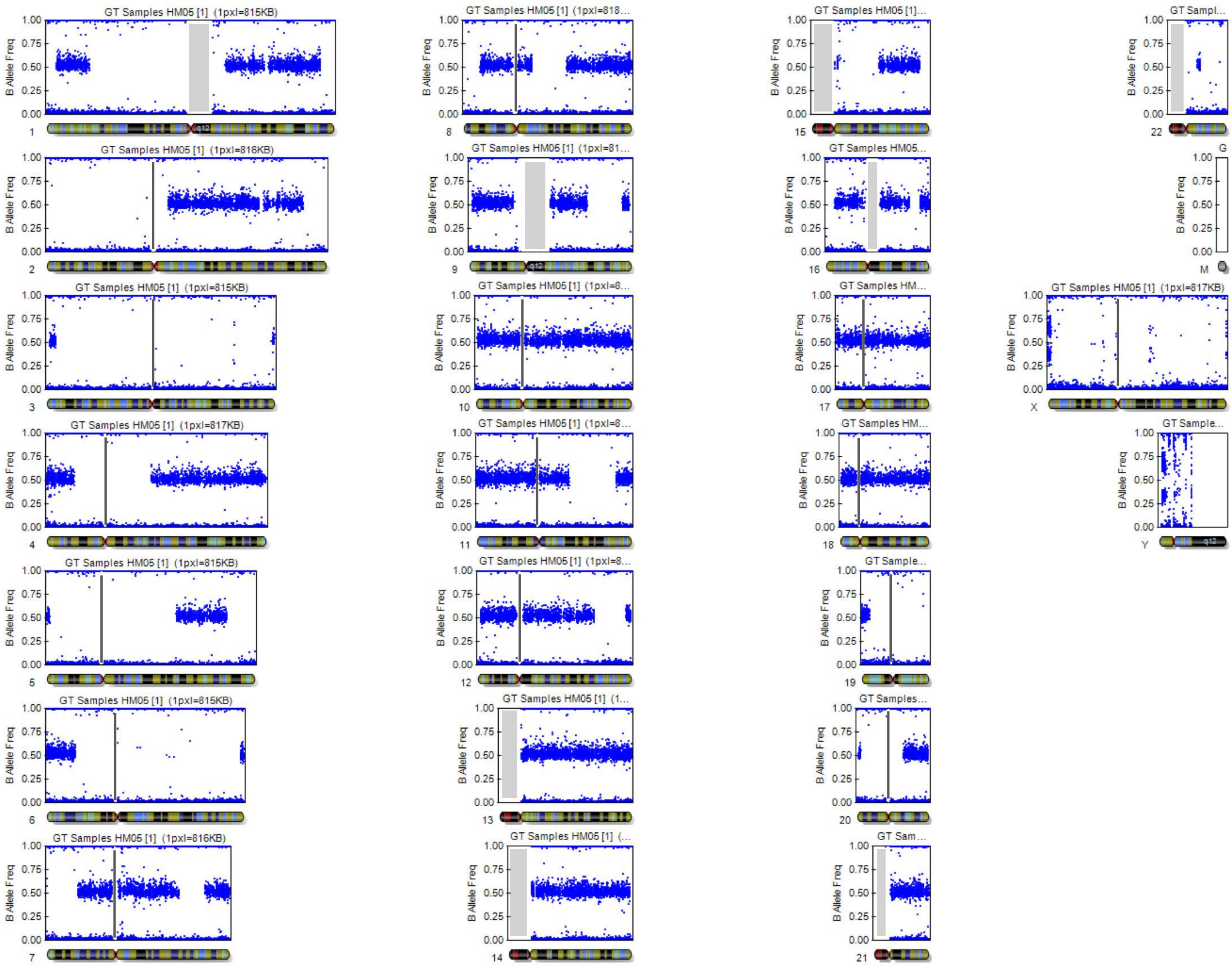
GT Samples HM05 [1] (1pxl=815KB)
GT Samples HM05 [1] (1pxl=816KB)
GT Samples HM05 [1] (1pxl=817KB)
B Allele Freq
1.00
0.75
0.50
0.25
0.00
1
2
3
4
5
6
7
8
9
10
11
12
13
14
15
16
17
18
19
20
21
22
X
Y
M
G
q12

HM05

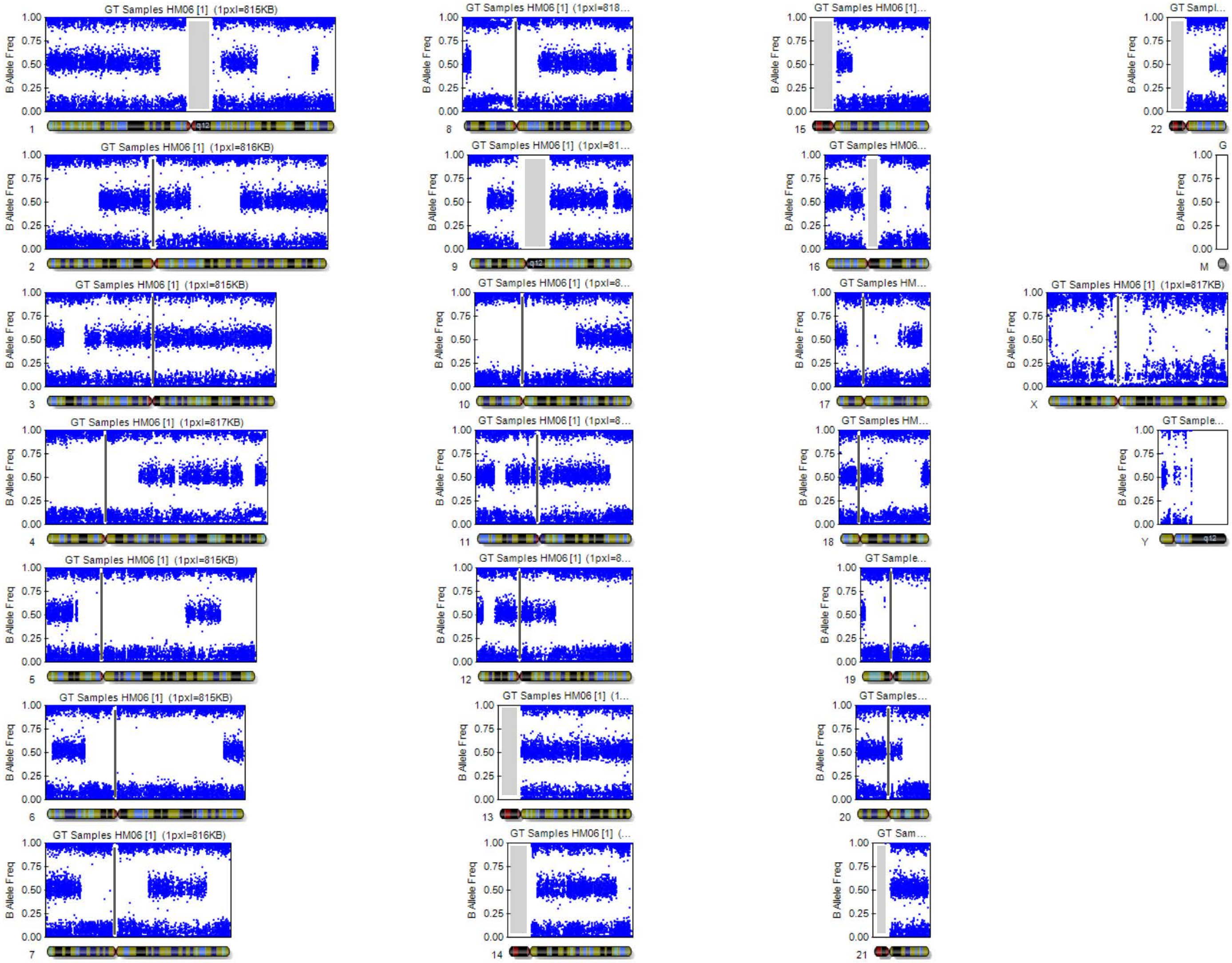
GT Samples HM06 [1] (1pxl=815KB)
GT Samples HM06 [1] (1pxl=816KB)
GT Samples HM06 [1] (1pxl=817KB)
B Allele Freq
1.00
0.75
0.50
0.25
0.00
1
2
3
4
5
6
7
8
9
10
11
12
13
14
15
16
17
18
19
20
21
22
X
Y
M
G

HM06

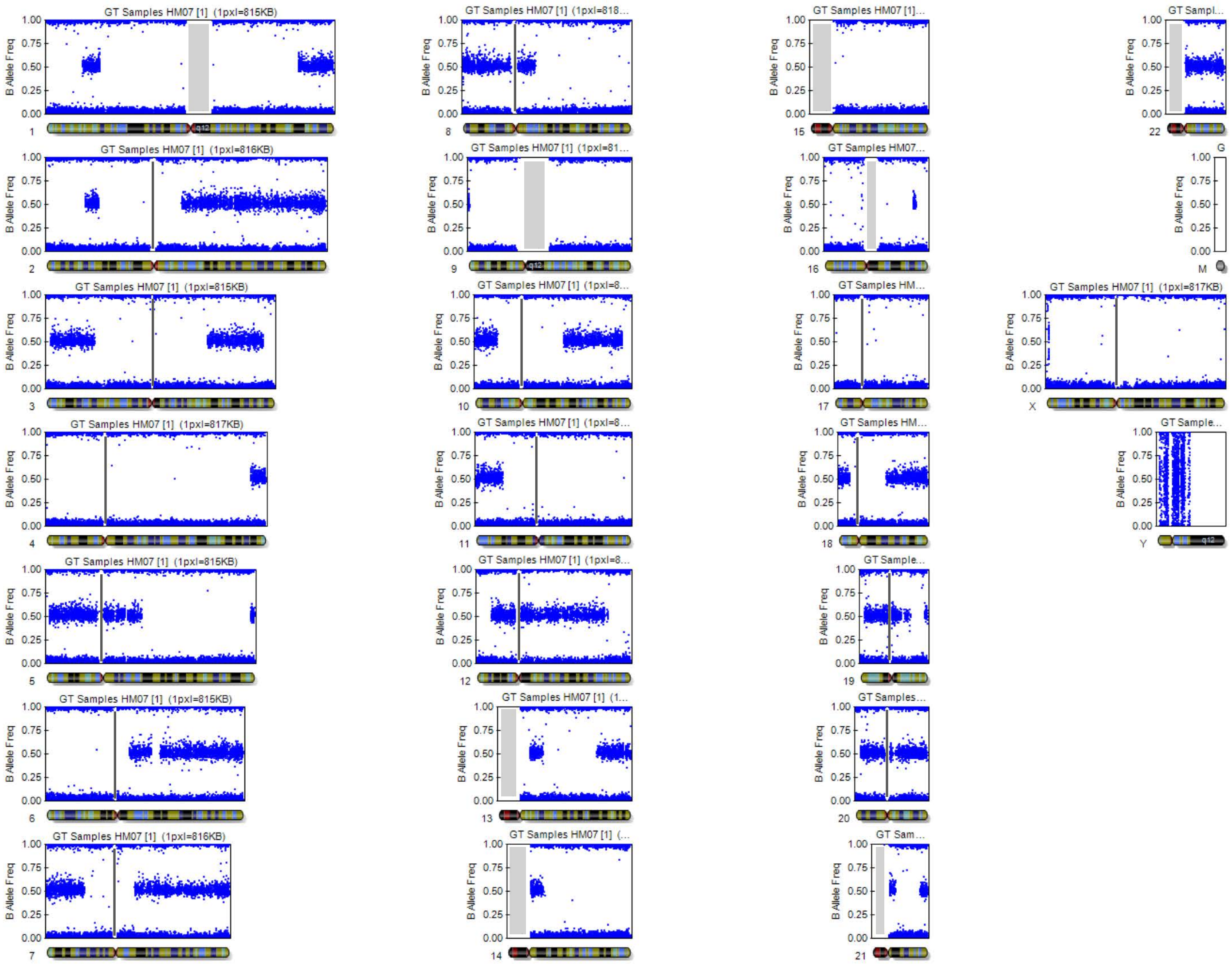
GT Samples HM07 [1] (1pxl=815KB)
GT Samples HM07 [1] (1pxl=816KB)
GT Samples HM07 [1] (1pxl=817KB)
B Allele Freq
1.00
0.75
0.50
0.25
0.00
1
2
3
4
5
6
7
8
9
10
11
12
13
14
15
16
17
18
19
20
21
22
X
Y
M
G

HM07

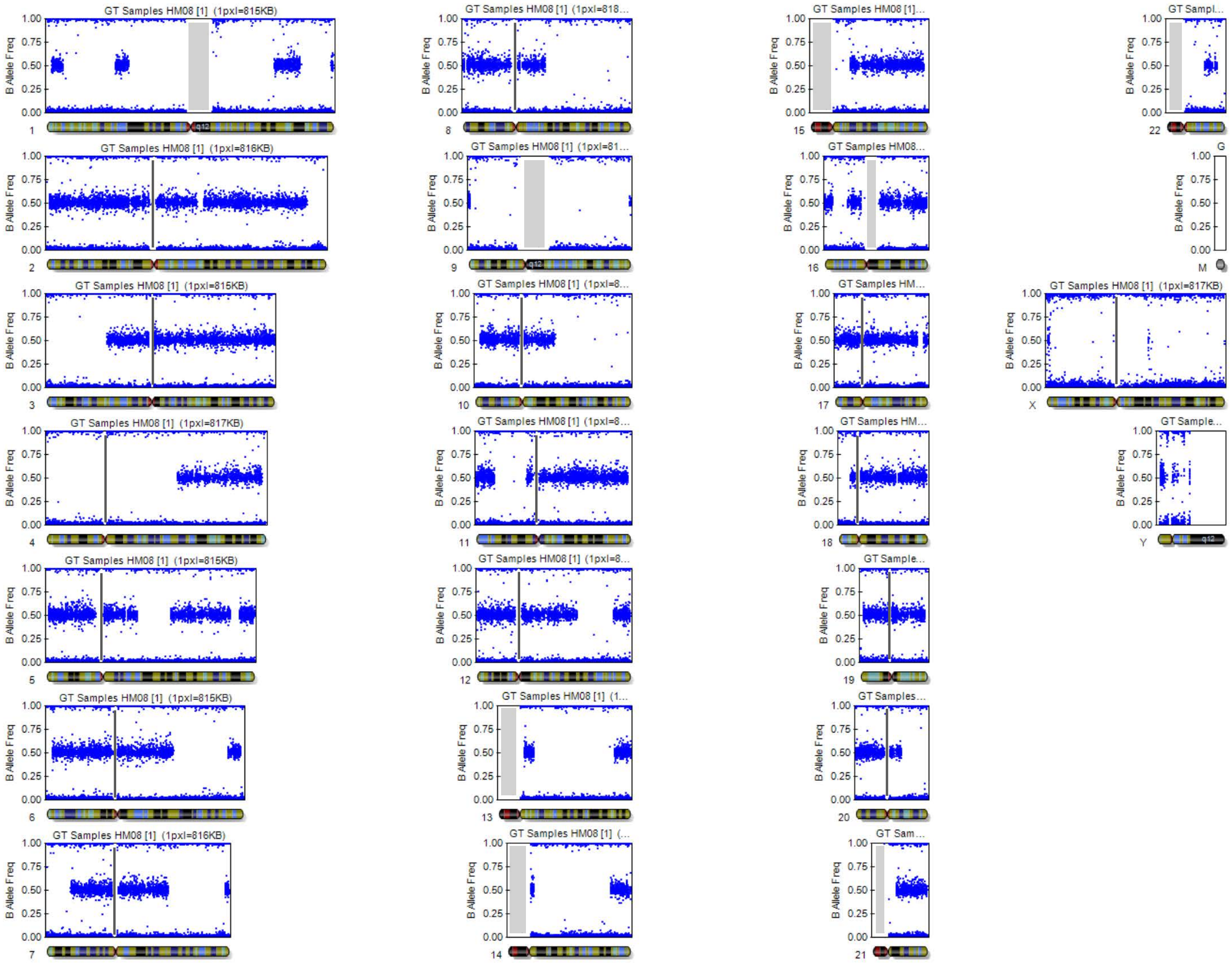
GT Samples HM08 [1] (1pxl=815KB)
GT Samples HM08 [1] (1pxl=816KB)
GT Samples HM08 [1] (1pxl=815KB)
GT Samples HM08 [1] (1pxl=817KB)
GT Samples HM08 [1] (1pxl=815KB)
GT Samples HM08 [1] (1pxl=815KB)
GT Samples HM08 [1] (1pxl=816KB)
GT Samples HM08 [1] (1pxl=817KB)
B Allele Freq
1.00
0.75
0.50
0.25
0.00
1
2
3
4
5
6
7
8
9
10
11
12
13
14
15
16
17
18
19
20
21
22
X
Y
M
G
q12

HM08

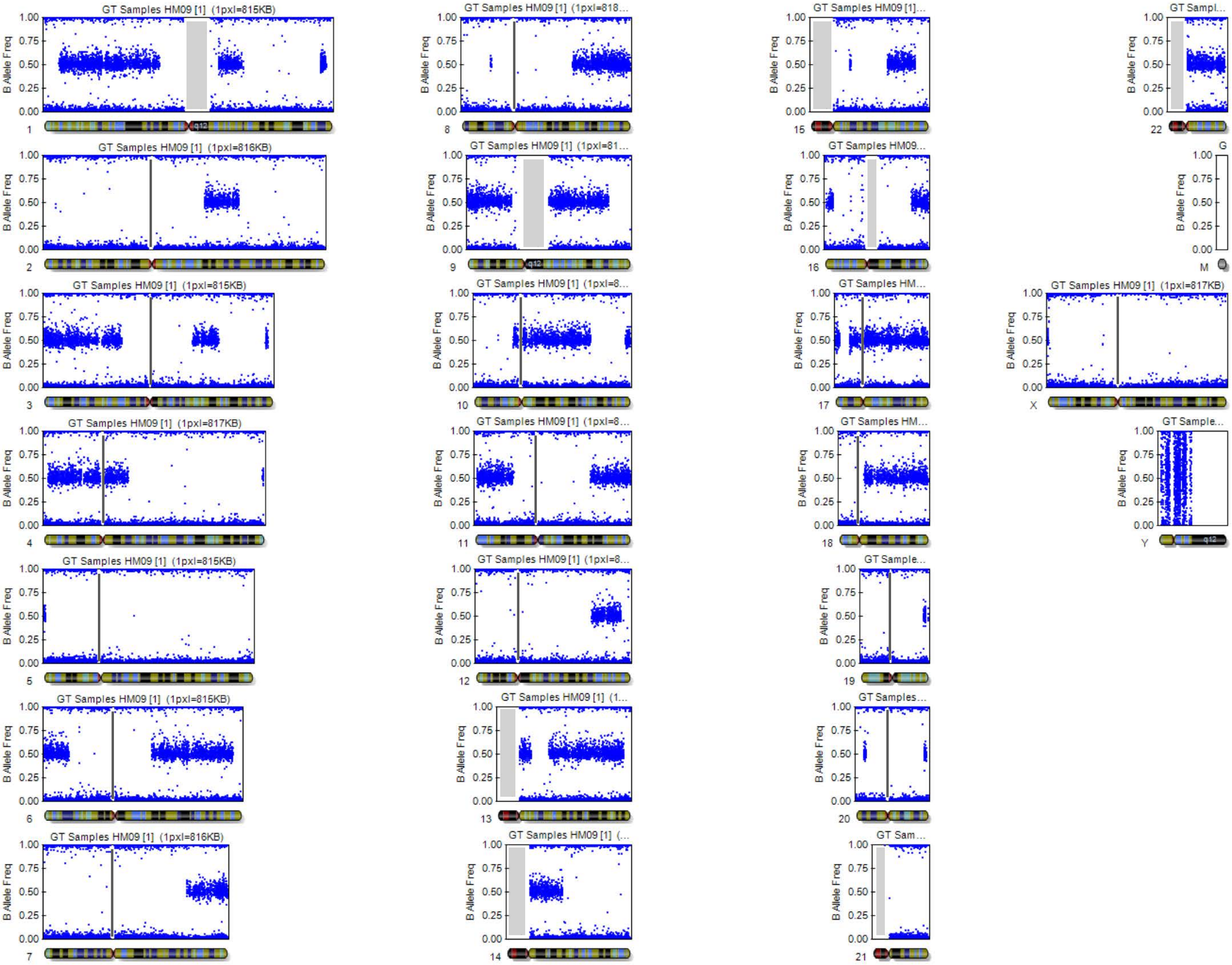
GT Samples HM09 [1] (1pxl=815KB)
GT Samples HM09 [1] (1pxl=816KB)
GT Samples HM09 [1] (1pxl=817KB)
B Allele Freq
1.00
0.75
0.50
0.25
0.00
1
2
3
4
5
6
7
8
9
10
11
12
13
14
15
16
17
18
19
20
21
22
M
X
Y
G
q12

HM09

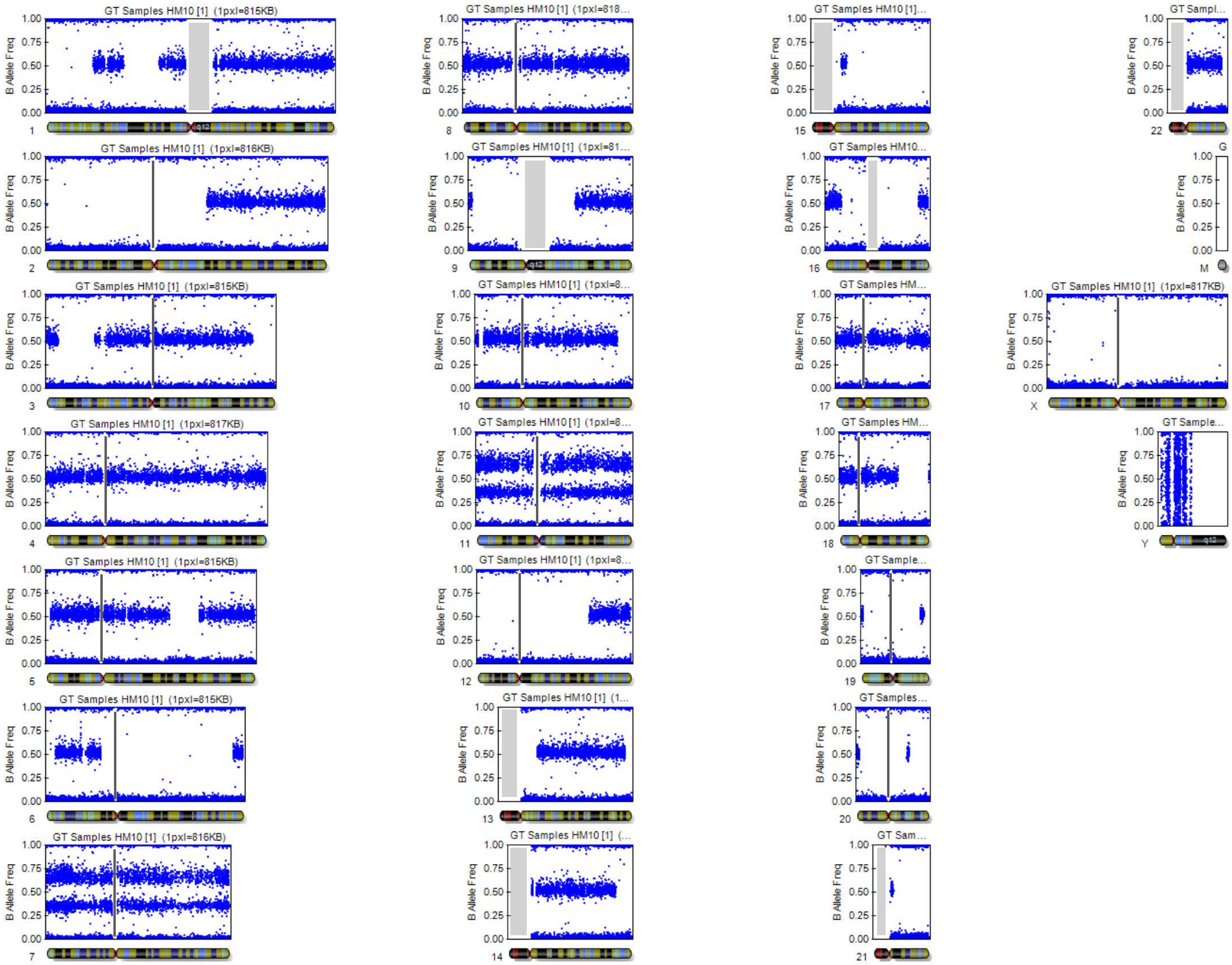
GT Samples HM10 [1] (1pxl=815KB)
GT Samples HM10 [1] (1pxl=816KB)
GT Samples HM10 [1] (1pxl=817KB)
B Allele Freq
1.00
0.75
0.50
0.25
0.00
1
2
3
4
5
6
7
8
9
10
11
12
13
14
15
16
17
18
19
20
21
22
X
Y
M
G
q12

HM10

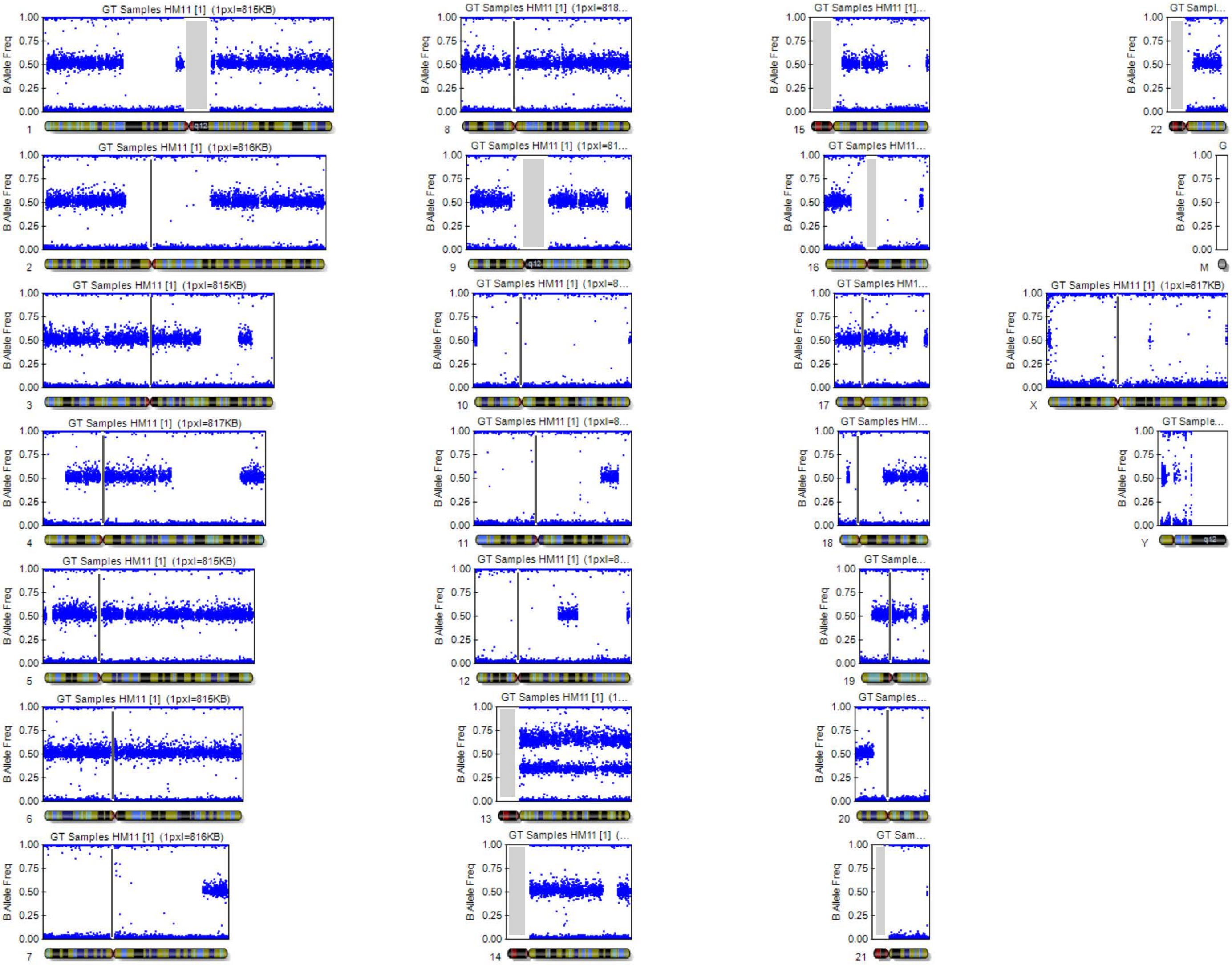
GT Samples HM11 [1] (1pxl=815KB)
GT Samples HM11 [1] (1pxl=816KB)
GT Samples HM11 [1] (1pxl=815KB)
GT Samples HM11 [1] (1pxl=817KB)
GT Samples HM11 [1] (1pxl=815KB)
GT Samples HM11 [1] (1pxl=815KB)
GT Samples HM11 [1] (1pxl=816KB)
GT Samples HM11 [1] (1pxl=818...
GT Samples HM11 [1] (1pxl=81...
GT Samples HM11 [1] (1pxl=8...
GT Samples HM11 [1] (1pxl=8...
GT Samples HM11 [1] (1pxl=8...
GT Samples HM11 [1] (1...
GT Samples HM11 [1] (...
GT Samples HM11 [1]...
GT Samples HM11...
GT Samples HM1...
GT Samples HM...
GT Sample...
GT Samples...
GT Sam...
GT Sampl...
GT Samples HM11 [1] (1pxl=817KB)
GT Sample...
B Allele Freq
1.00
0.75
0.50
0.25
0.00
1
2
3
4
5
6
7
8
9
10
11
12
13
14
15
16
17
18
19
20
21
22
G
M
X
Y

HM11

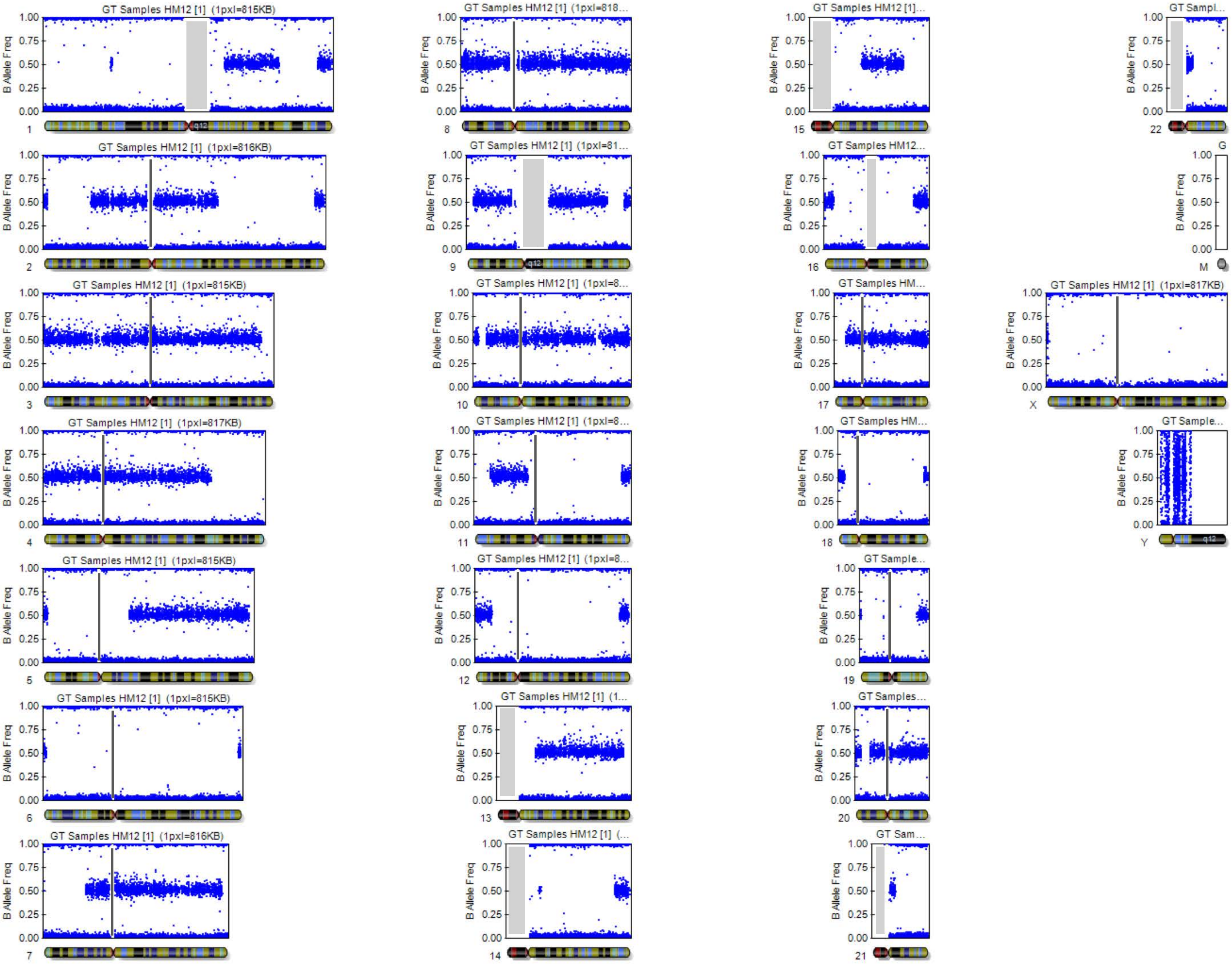

GT Samples HM12 [1] (1pxl=815KB)
GT Samples HM12 [1] (1pxl=816KB)
GT Samples HM12 [1] (1pxl=817KB)
B Allele Freq
1.00
0.75
0.50
0.25
0.00
1
2
3
4
5
6
7
8
9
10
11
12
13
14
15
16
17
18
19
20
21
22
X
Y
M
G

HM12

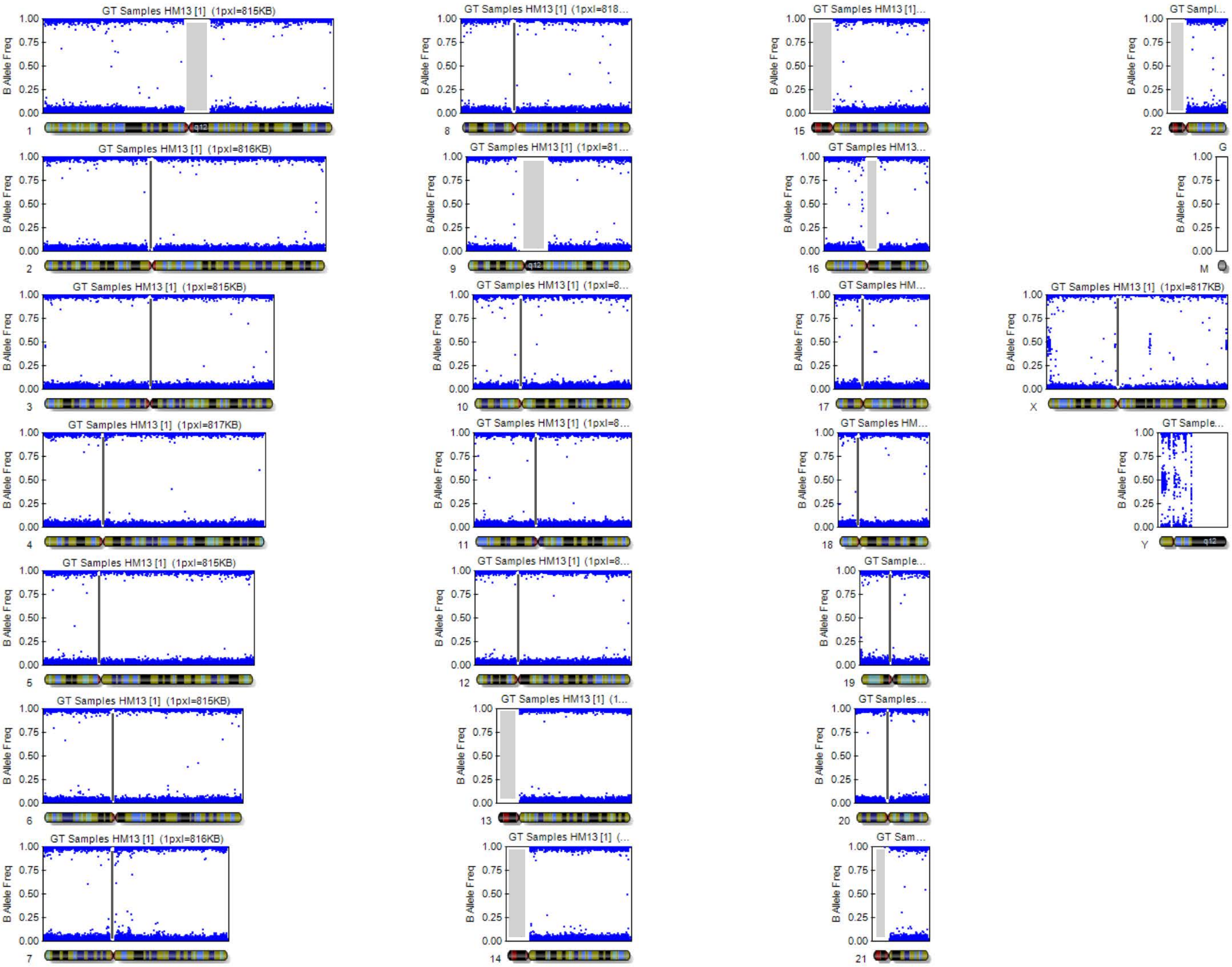
GT Samples HM13 [1] (1pxl=815KB)
GT Samples HM13 [1] (1pxl=816KB)
GT Samples HM13 [1] (1pxl=817KB)
B Allele Freq
1.00
0.75
0.50
0.25
0.00
1
2
3
4
5
6
7
8
9
10
11
12
13
14
15
16
17
18
19
20
21
22
X
Y
M
G
q12

HM13

Supplementary Figure S2:

Log R ratio plotting of all heterozygous androgenetic complete hydatidiform moles. Blue dots indicate the log R ratio values calculated from the R and θ value. The red line in the log R ratio plot indicates a smoothing series with a 200kb moving average window.

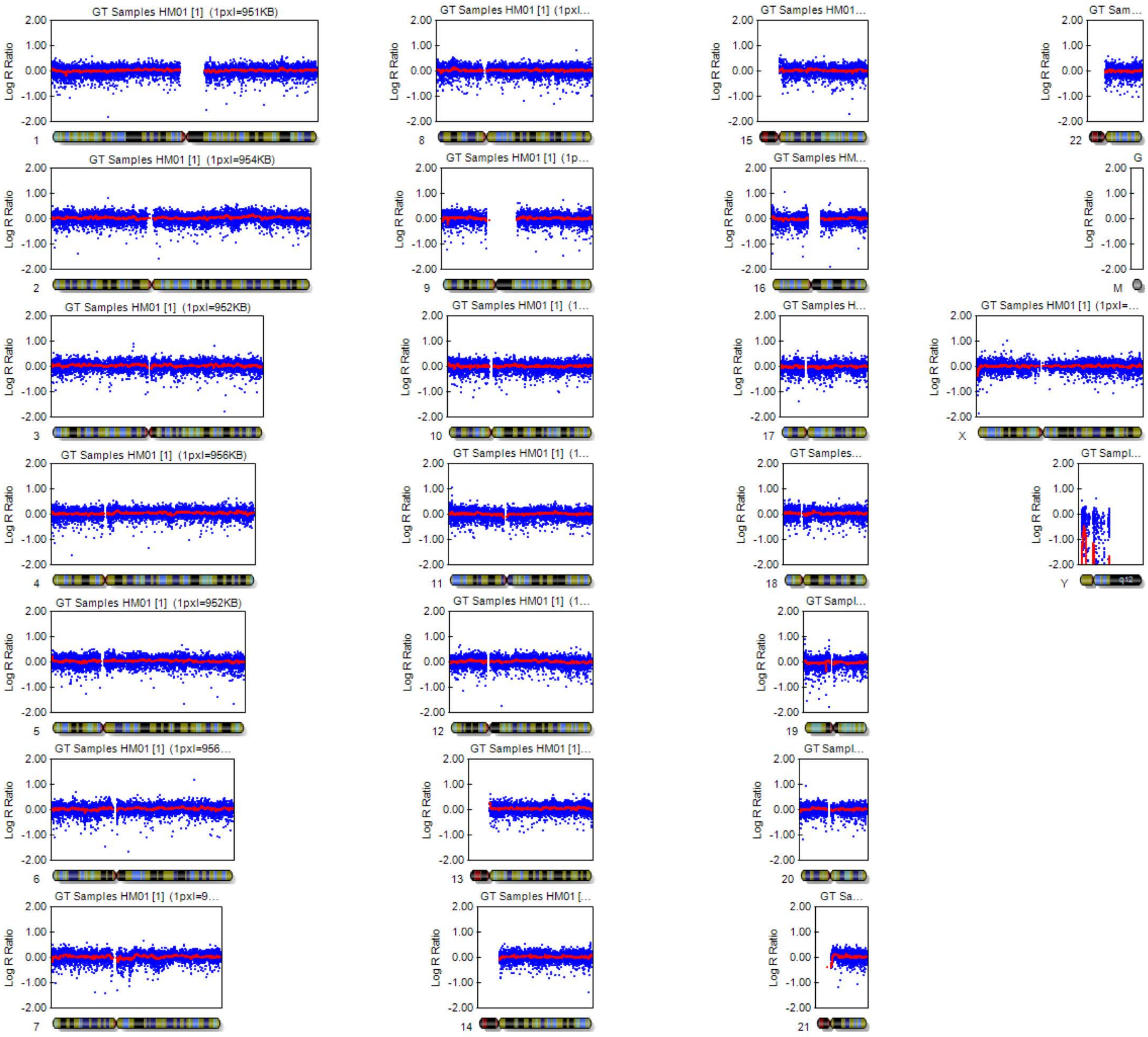
GT Samples HM01 [1] (1pxl=951KB)
GT Samples HM01 [1] (1pxl=954KB)
GT Samples HM01 [1] (1pxl=952KB)
GT Samples HM01 [1] (1pxl=956KB)
GT Samples HM01 [1] (1pxl=952KB)
Log R Ratio

HM01

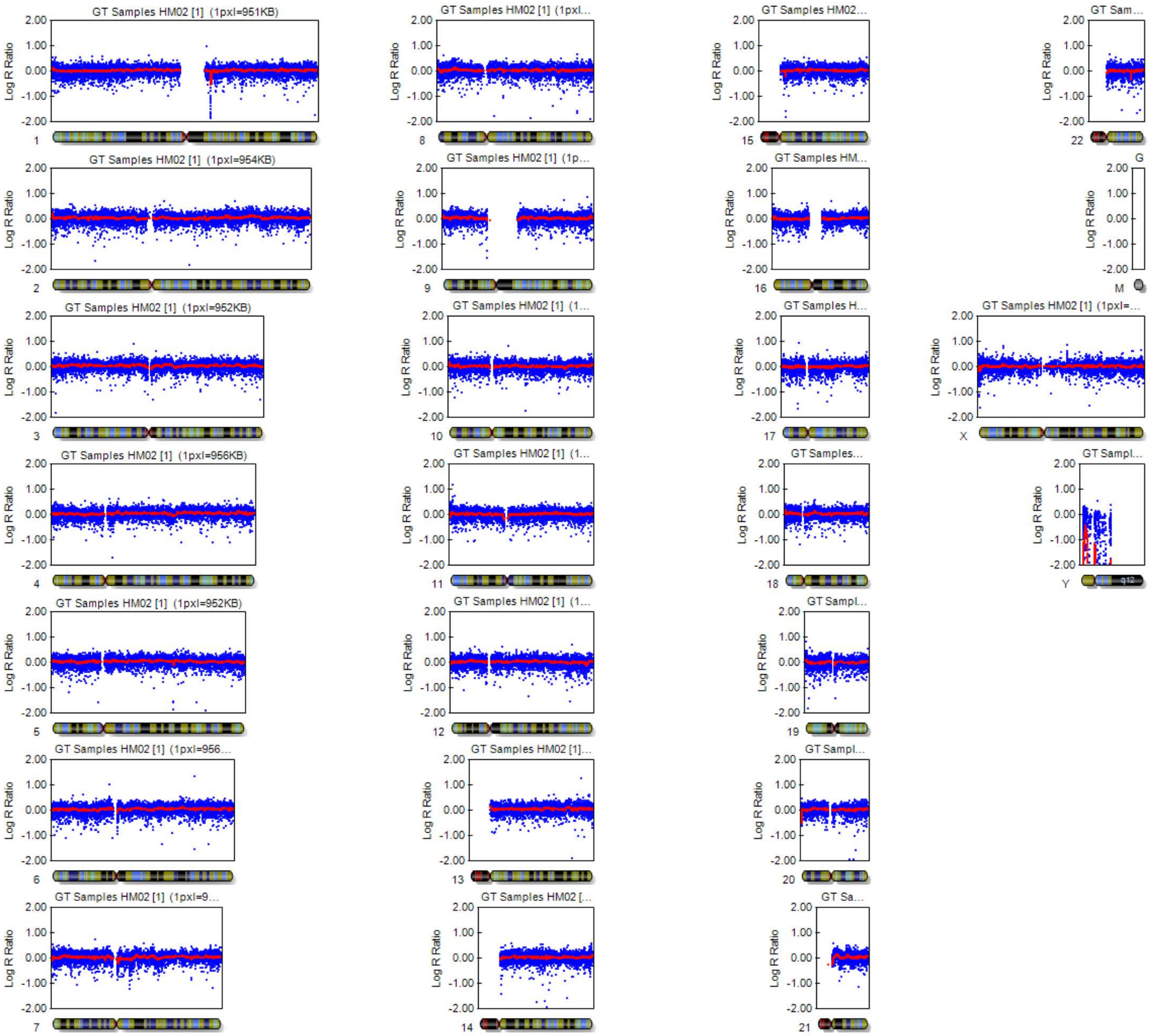
GT Samples HM02 [1] (1pxl=951KB)
GT Samples HM02 [1] (1pxl=954KB)
GT Samples HM02 [1] (1pxl=952KB)
GT Samples HM02 [1] (1pxl=956KB)
GT Samples HM02 [1] (1pxl=952KB)
Log R Ratio
2.00
1.00
0.00
-1.00
-2.00
1
2
3
4
5
6
7
8
9
10
11
12
13
14
15
16
17
18
19
20
21
22
X
Y
M
G
q12

HM02

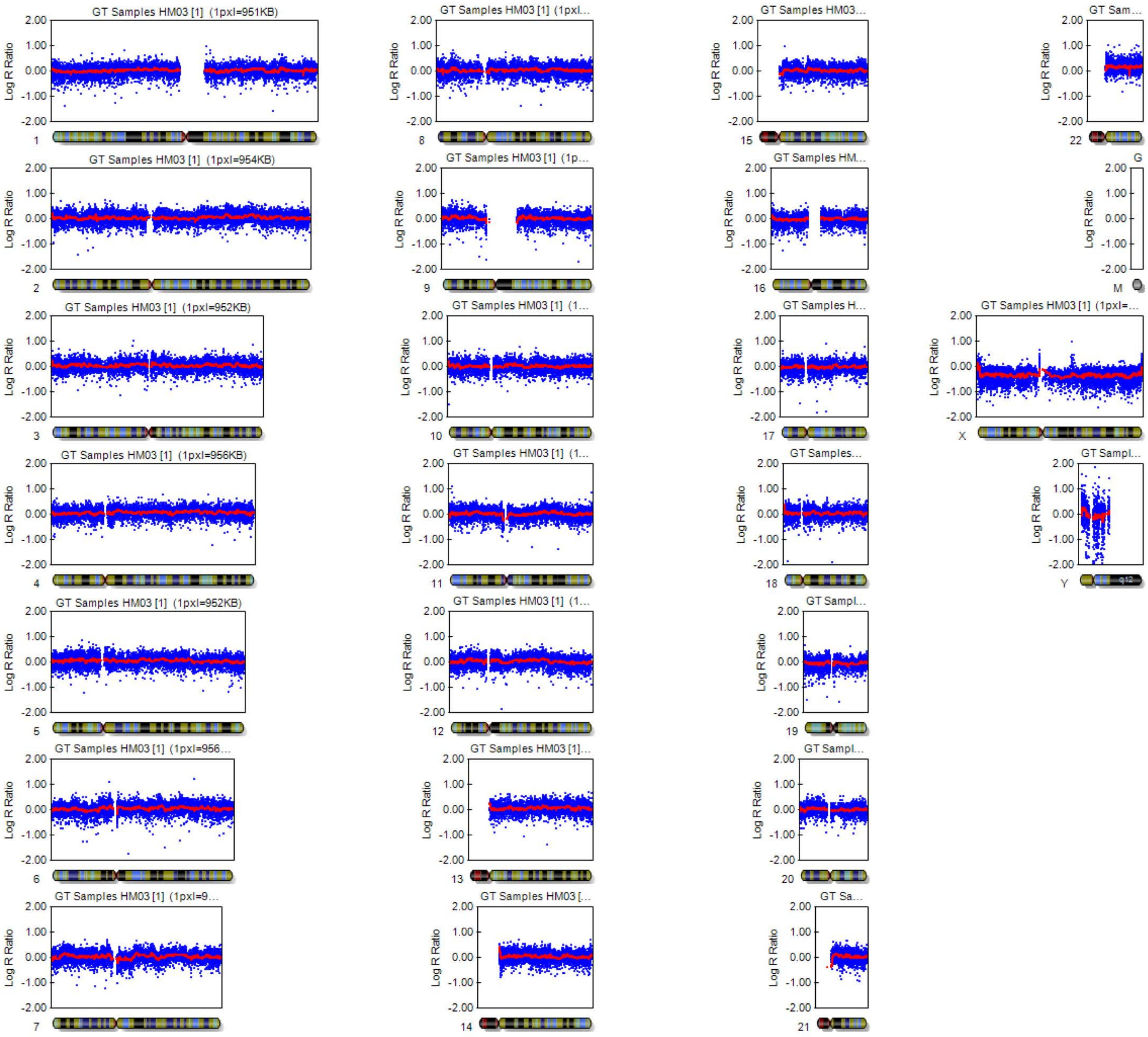
GT Samples HM03 [1] (1pxl=951KB)
GT Samples HM03 [1] (1pxl=954KB)
GT Samples HM03 [1] (1pxl=952KB)
GT Samples HM03 [1] (1pxl=956KB)
GT Samples HM03 [1] (1pxl=952KB)
Log R Ratio
2.00
1.00
0.00
-1.00
-2.00
1
2
3
4
5
6
7
8
9
10
11
12
13
14
15
16
17
18
19
20
21
22
X
Y
M
G
q12

HM03

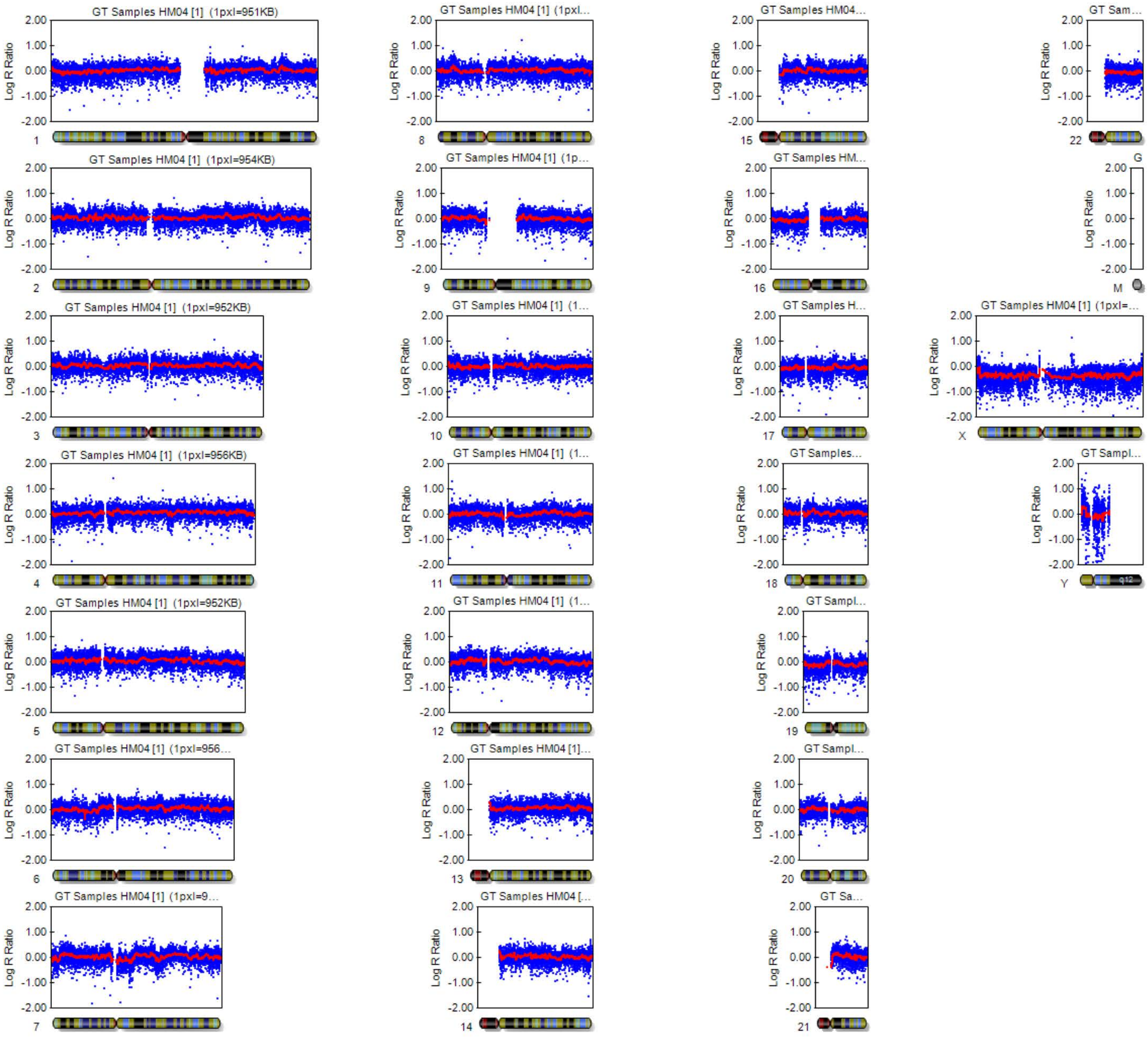
GT Samples HM04 [1] (1pxl=951KB)
GT Samples HM04 [1] (1pxl=954KB)
GT Samples HM04 [1] (1pxl=952KB)
GT Samples HM04 [1] (1pxl=956KB)
GT Samples HM04 [1] (1pxl=952KB)
GT Samples HM04 [1] (1pxl=956...
GT Samples HM04 [1] (1pxl=9...
GT Samples HM04 [1] (1pxl...
GT Samples HM04 [1] (1p...
GT Samples HM04 [1] (1...
GT Samples HM04 [1] (1...
GT Samples HM04 [1] (1...
GT Samples HM04 [1]...
GT Samples HM04 [...
GT Samples HM04...
GT Samples HM...
GT Samples H...
GT Samples...
GT Sampl...
GT Sampl...
GT Sa...
GT Sam...
GT Samples HM04 [1] (1pxl=...
GT Sampl...
Log R Ratio
2.00
1.00
0.00
-1.00
-2.00
1
2
3
4
5
6
7
8
9
10
11
12
13
14
15
16
17
18
19
20
21
22
G
M
X
Y
q12

HM04

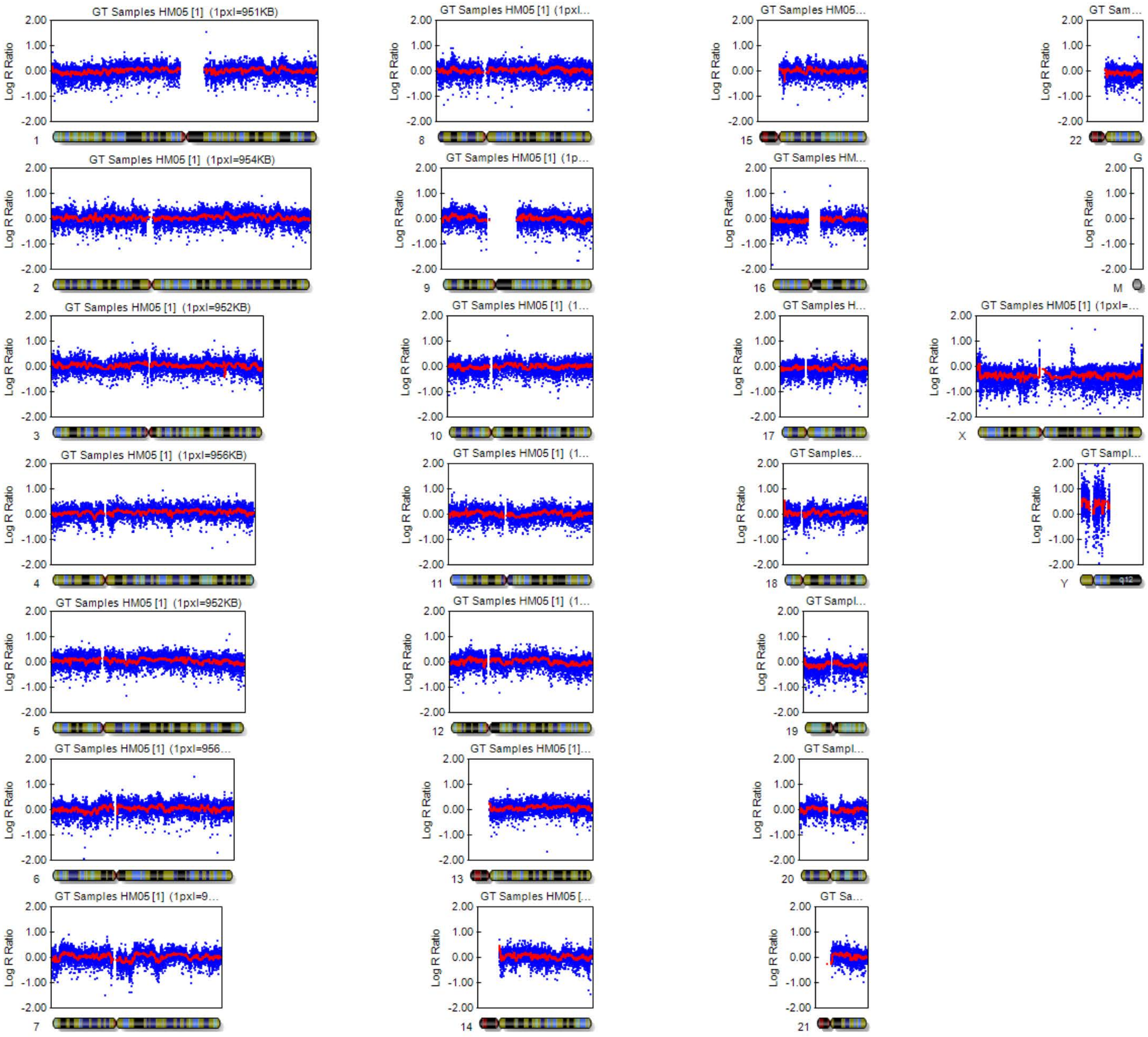
GT Samples HM05 [1] (1pxl=951KB)
GT Samples HM05 [1] (1pxl=954KB)
GT Samples HM05 [1] (1pxl=952KB)
GT Samples HM05 [1] (1pxl=956KB)
GT Samples HM05 [1] (1pxl=952KB)
GT Samples HM05 [1] (1pxl=956...
GT Samples HM05 [1] (1pxl=9...
GT Samples HM05 [1] (1pxl...
GT Samples HM05 [1] (1p...
GT Samples HM05 [1] (1...
GT Samples HM05 [1]...
GT Samples HM05 [...
GT Samples HM05...
GT Samples HM...
GT Samples H...
GT Samples...
GT Sampl...
GT Sa...
GT Sam...
GT Samples HM05 [1] (1pxl=...
Log R Ratio
2.00
1.00
0.00
-1.00
-2.00
1
2
3
4
5
6
7
8
9
10
11
12
13
14
15
16
17
18
19
20
21
22
X
Y
M
G
q12

HM05

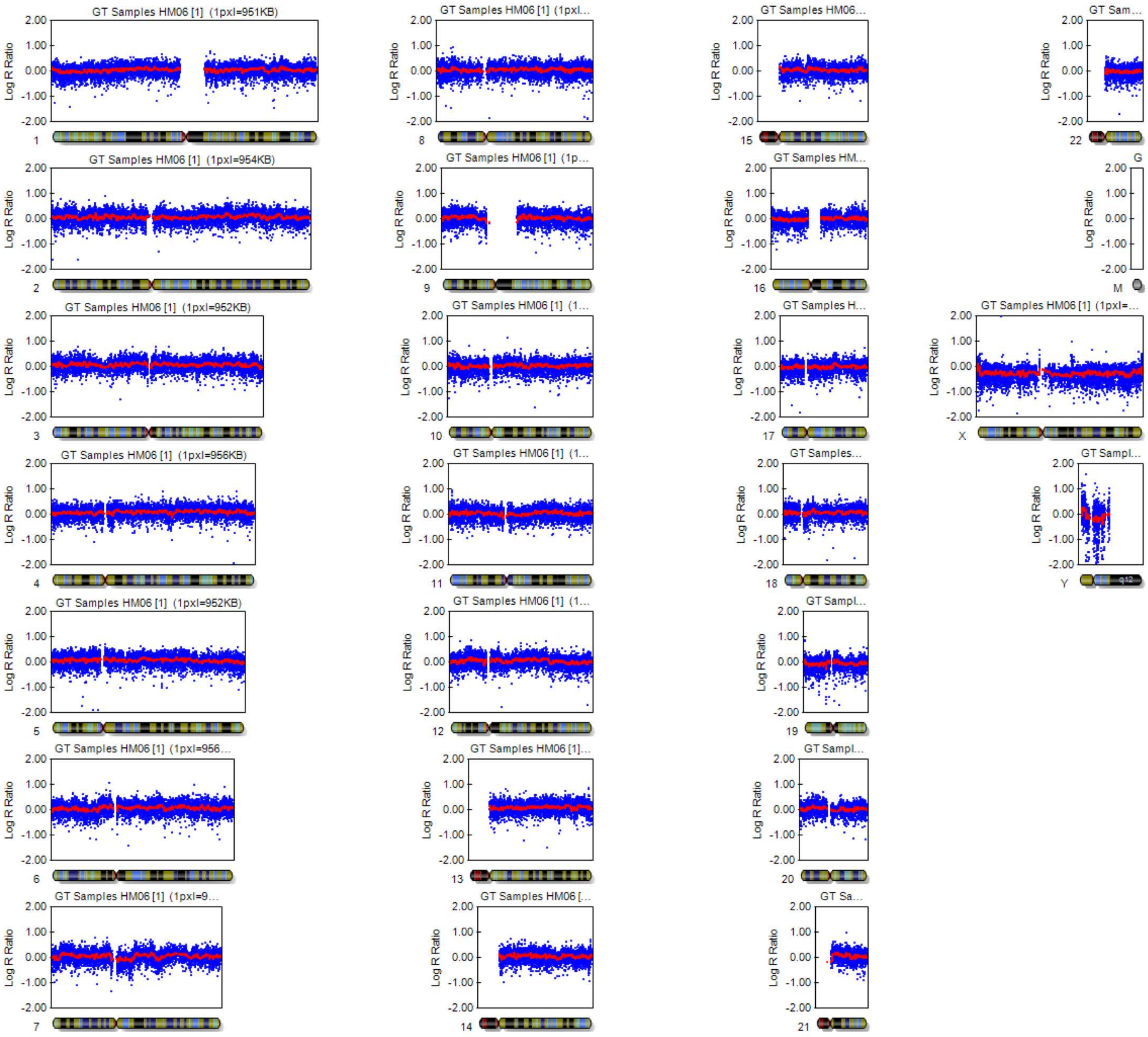
GT Samples HM06 [1] (1pxl=951KB)
GT Samples HM06 [1] (1pxl=954KB)
GT Samples HM06 [1] (1pxl=952KB)
GT Samples HM06 [1] (1pxl=956KB)
GT Samples HM06 [1] (1pxl=952KB)
GT Samples HM06 [1] (1pxl=956...
GT Samples HM06 [1] (1pxl=9...
Log R Ratio
2.00
1.00
0.00
-1.00
-2.00
1
2
3
4
5
6
7
8
9
10
11
12
13
14
15
16
17
18
19
20
21
22
X
Y
M
G
q12

HM06

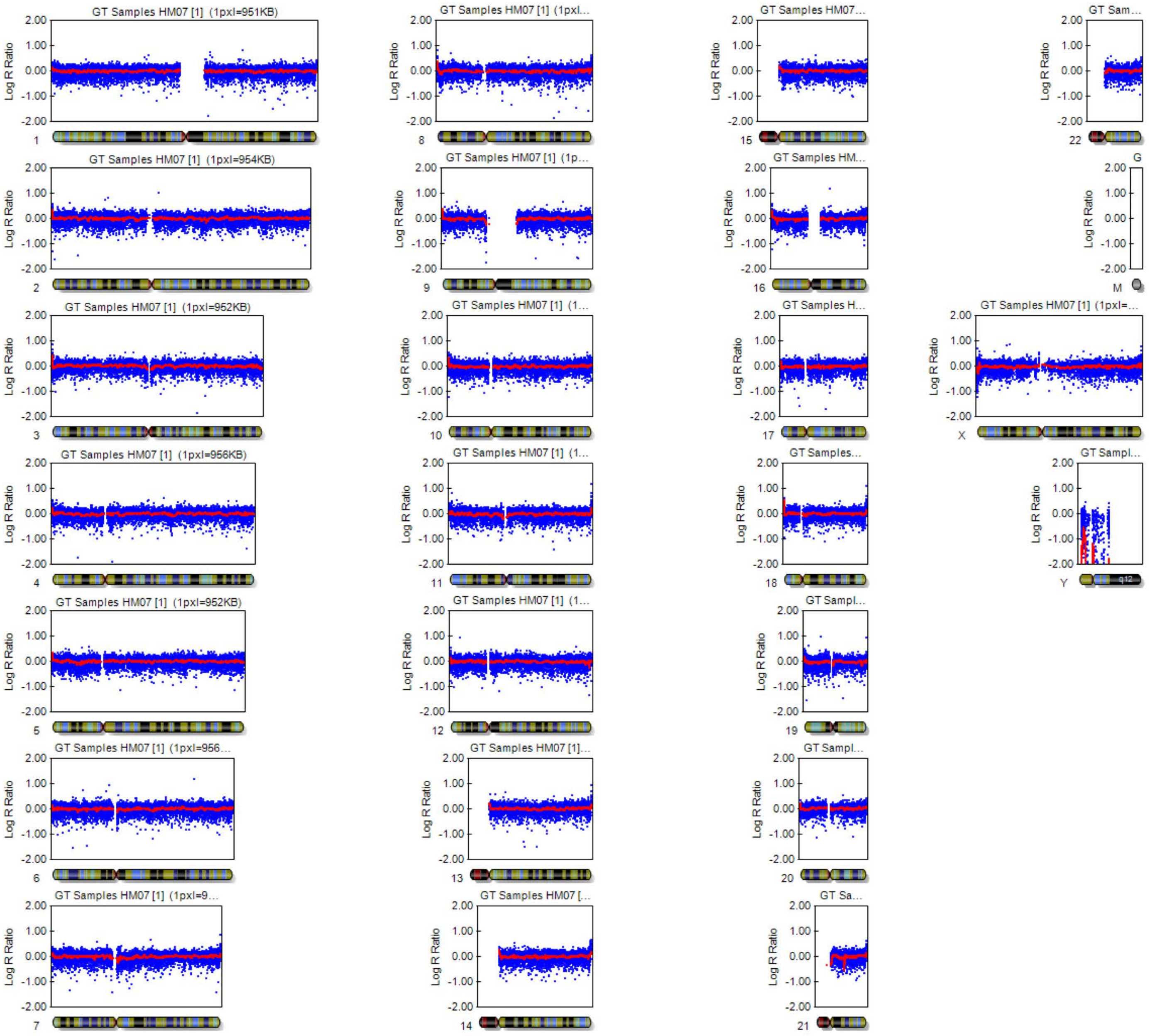
GT Samples HM07 [1] (1pxl=951KB)
1
GT Samples HM07 [1] (1pxl=954KB)
2
GT Samples HM07 [1] (1pxl=952KB)
3
GT Samples HM07 [1] (1pxl=956KB)
4
GT Samples HM07 [1] (1pxl=952KB)
5
GT Samples HM07 [1] (1pxl=956...
6
GT Samples HM07 [1] (1pxl=9...
7
GT Samples HM07 [1] (1pxl...
8
GT Samples HM07 [1] (1p...
9
GT Samples HM07 [1] (1...
10
GT Samples HM07 [1] (1...
11
GT Samples HM07 [1] (1...
12
GT Samples HM07 [1]...
13
GT Samples HM07 [...
14
GT Samples HM07...
15
GT Samples HM...
16
GT Samples H...
17
GT Samples...
18
GT Sampl...
19
GT Sampl...
20
GT Sa...
21
GT Sam...
22
G
M
GT Samples HM07 [1] (1pxl=...
X
GT Sampl...
Y
q12
Log R Ratio

HM07

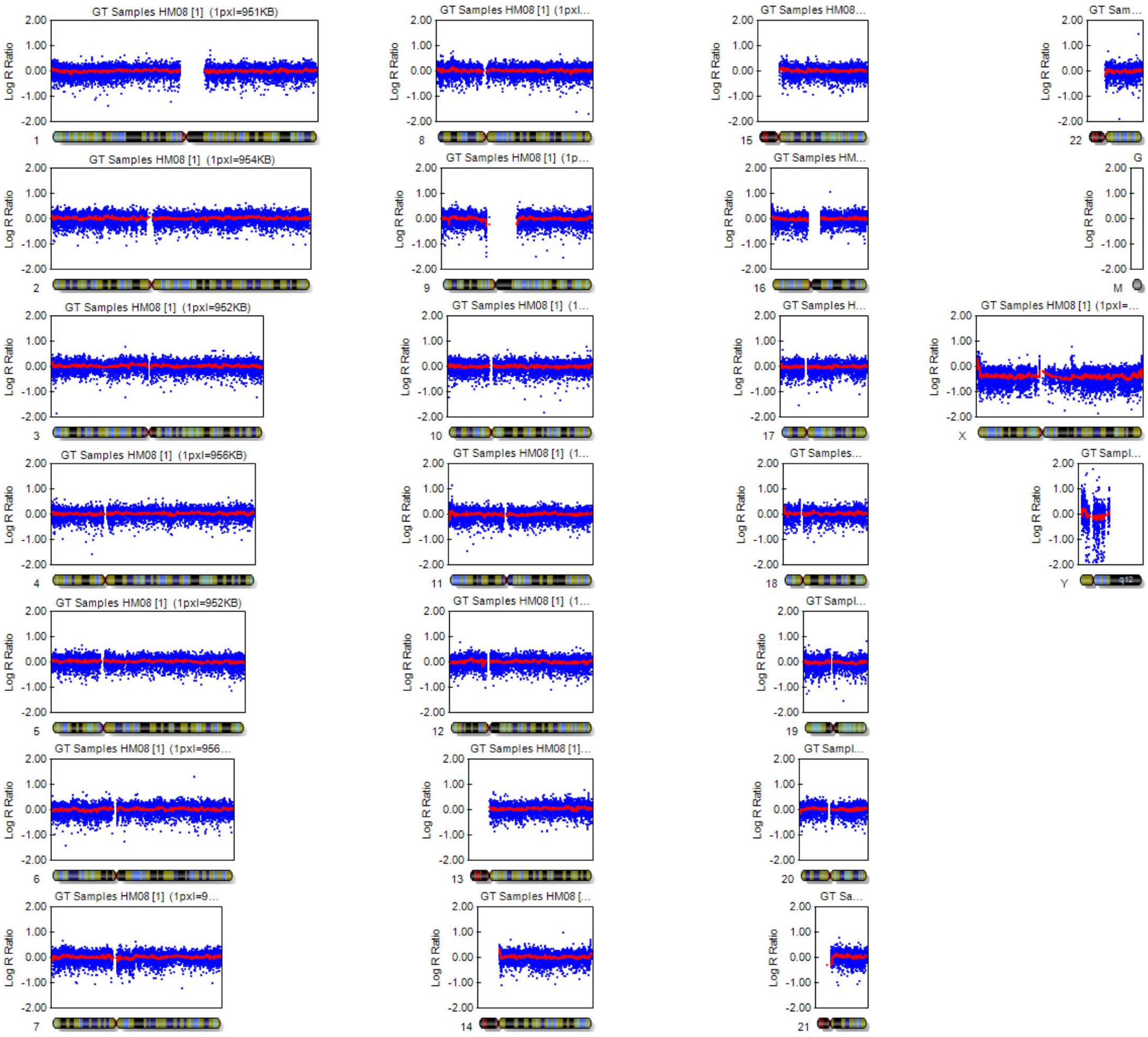

GT Samples HM08 [1] (1pxl=951KB)
GT Samples HM08 [1] (1pxl=954KB)
GT Samples HM08 [1] (1pxl=952KB)
GT Samples HM08 [1] (1pxl=956KB)
GT Samples HM08 [1] (1pxl=952KB)
Log R Ratio

HM08

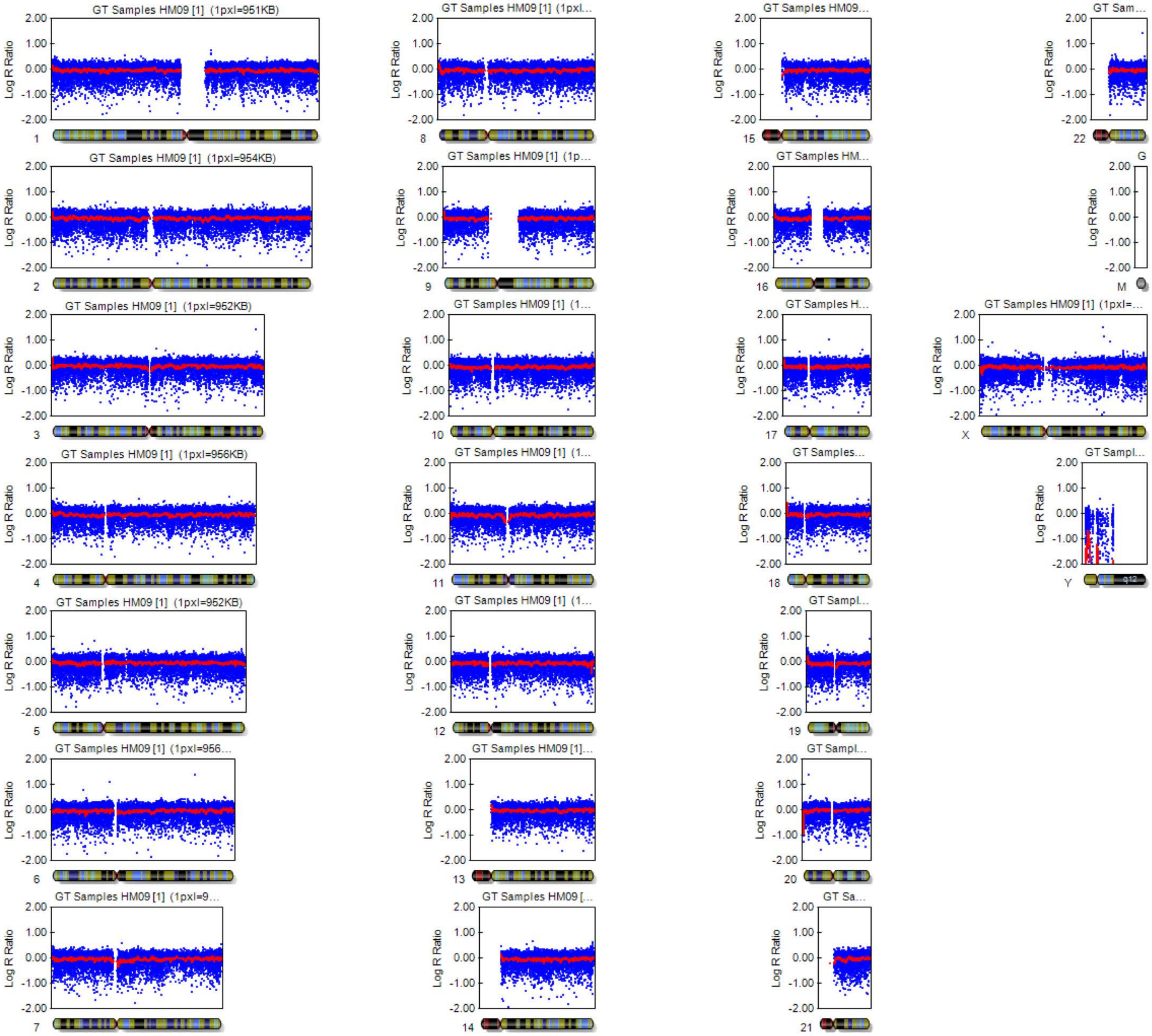
GT Samples HM09 [1] (1pxl=951KB)
GT Samples HM09 [1] (1pxl=954KB)
GT Samples HM09 [1] (1pxl=952KB)
GT Samples HM09 [1] (1pxl=956KB)
GT Samples HM09 [1] (1pxl=952KB)
Log R Ratio
2.00
1.00
0.00
-1.00
-2.00
1
2
3
4
5
6
7
8
9
10
11
12
13
14
15
16
17
18
19
20
21
22
M
X
Y
q12

HM09

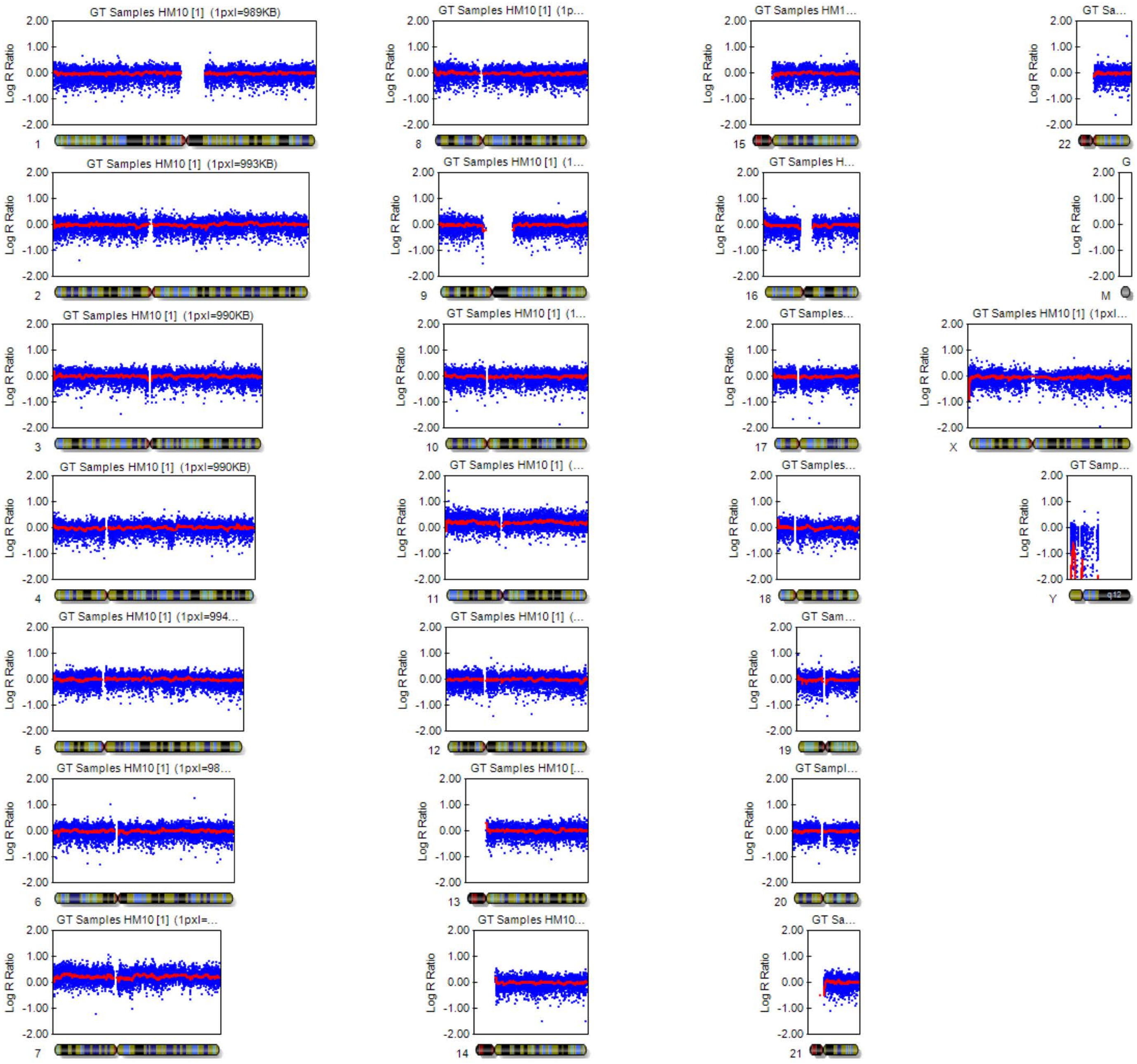
GT Samples HM10 [1] (1pxl=989KB)
GT Samples HM10 [1] (1pxl=993KB)
GT Samples HM10 [1] (1pxl=990KB)
GT Samples HM10 [1] (1pxl=990KB)
GT Samples HM10 [1] (1pxl=994...
GT Samples HM10 [1] (1pxl=98...
GT Samples HM10 [1] (1pxl=...
Log R Ratio
2.00
1.00
0.00
-1.00
-2.00
1
2
3
4
5
6
7
8
9
10
11
12
13
14
15
16
17
18
19
20
21
22
X
Y
M
G
q12

HM10

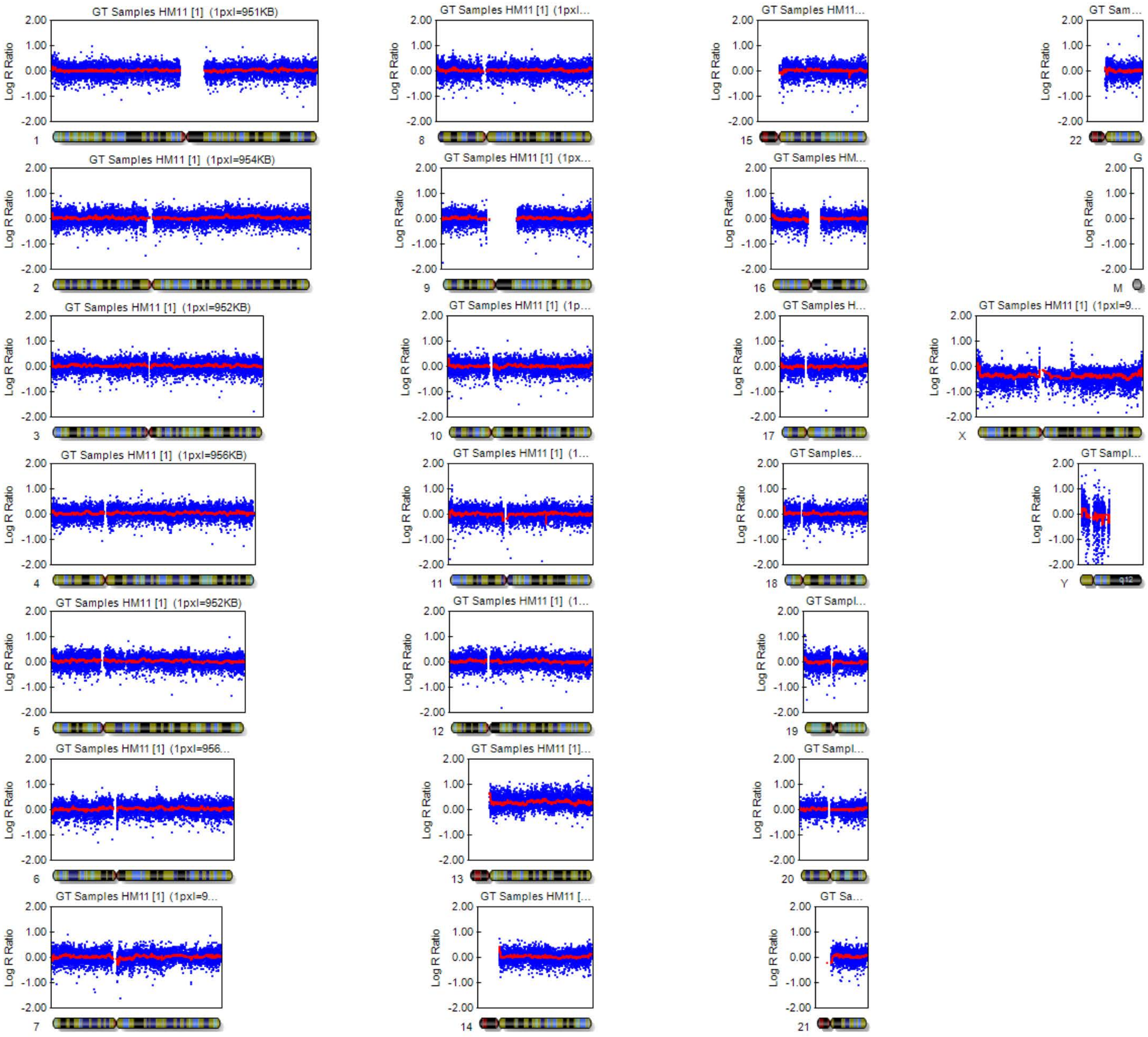
GT Samples HM11 [1] (1pxl=951KB)
GT Samples HM11 [1] (1pxl=954KB)
GT Samples HM11 [1] (1pxl=952KB)
GT Samples HM11 [1] (1pxl=956KB)
GT Samples HM11 [1] (1pxl=952KB)
Log R Ratio
2.00
1.00
0.00
-1.00
-2.00
1
2
3
4
5
6
7
8
9
10
11
12
13
14
15
16
17
18
19
20
21
22
X
Y
M
G
q12

HM11

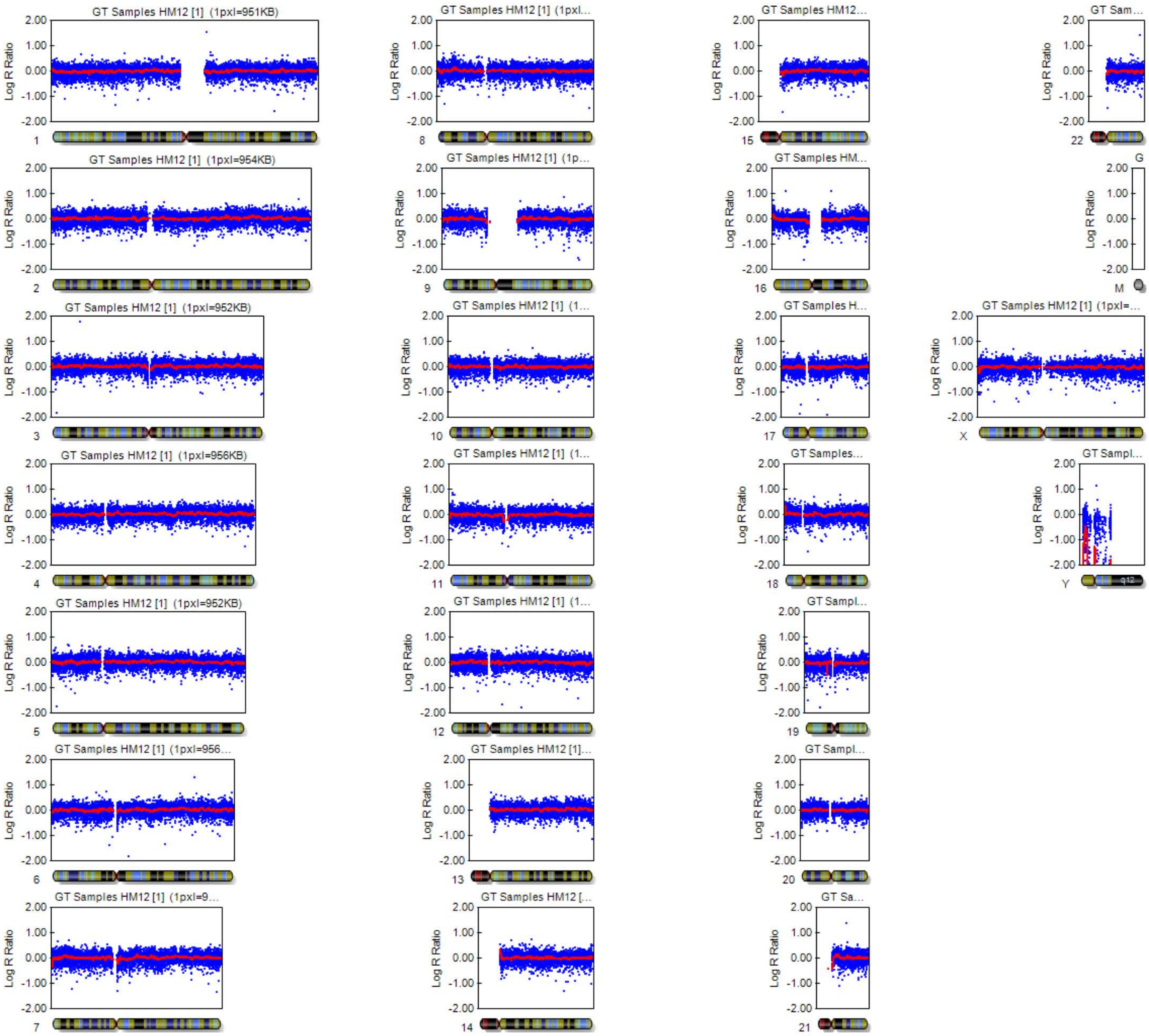
GT Samples HM12 [1] (1pxl=951KB)
Log R Ratio
1
8
15
22
2
9
16
M
3
10
17
X
4
11
18
Y
5
12
19
6
13
20
7
14
21

HM12

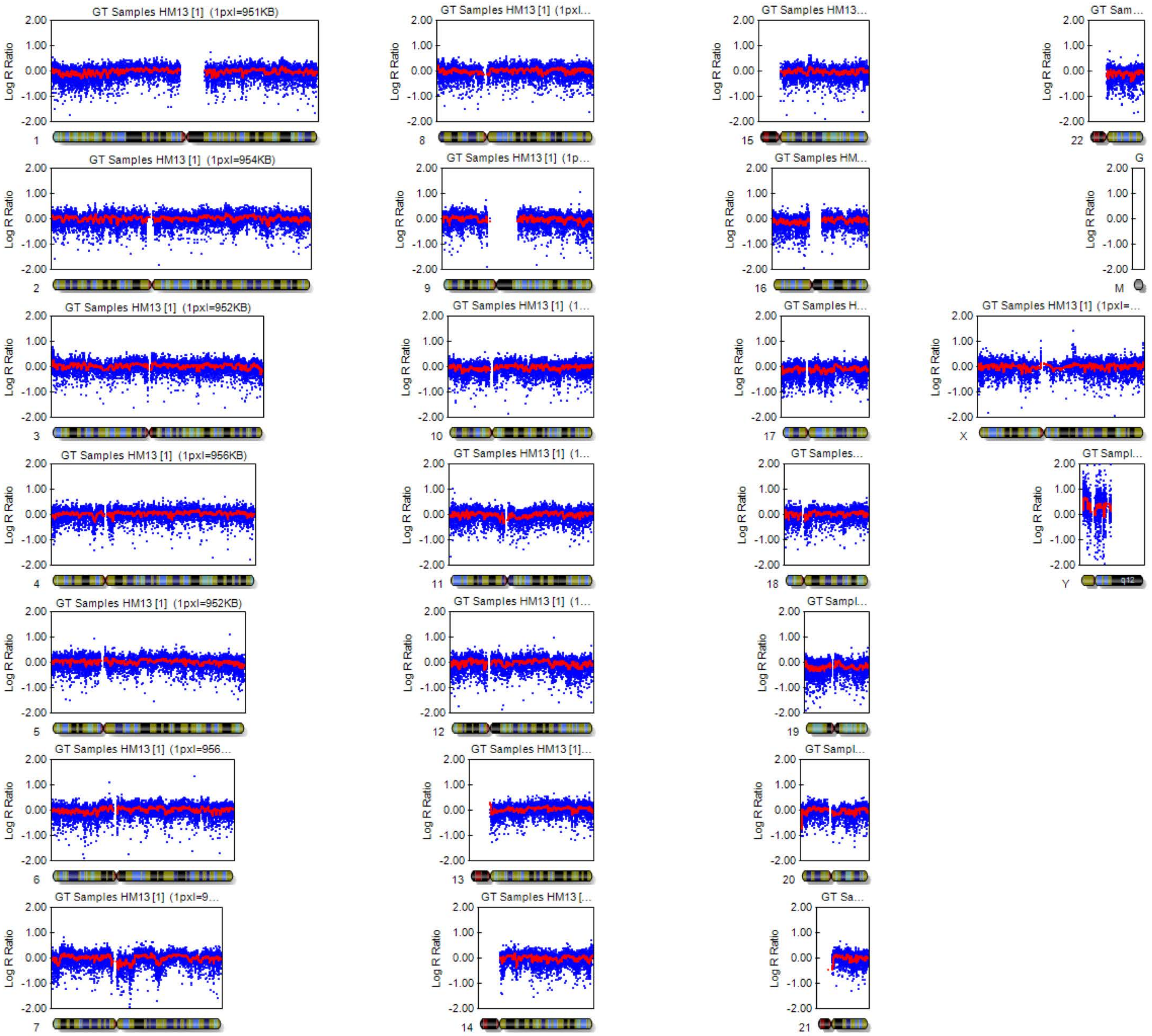

GT Samples HM13 [1] (1pxl=951KB)
GT Samples HM13 [1] (1pxl=954KB)
GT Samples HM13 [1] (1pxl=952KB)
GT Samples HM13 [1] (1pxl=956KB)
GT Samples HM13 [1] (1pxl=952KB)
GT Samples HM13 [1] (1pxl=956...
GT Samples HM13 [1] (1pxl=9...
GT Samples HM13 [1] (1pxl...
GT Samples HM13 [1] (1p...
GT Samples HM13 [1] (1...
GT Samples HM13 [1] (1...
GT Samples HM13 [1] (1...
GT Samples HM13 [1]...
GT Samples HM13 [...
GT Samples HM13...
GT Samples HM...
GT Samples H...
GT Samples...
GT Sampl...
GT Sampl...
GT Sa...
GT Sam...
G
GT Samples HM13 [1] (1pxl=...
GT Sampl...
Log R Ratio
2.00
1.00
0.00
-1.00
-2.00
1
2
3
4
5
6
7
8
9
10
11
12
13
14
15
16
17
18
19
20
21
22
M
X
Y
q12

HM13

Supplementary Figure S3:

Distribution of log R ratio of chromosomes 7, 11, 13, and 22. Histograms were prepared using the R software (https://www.R-project.org/).

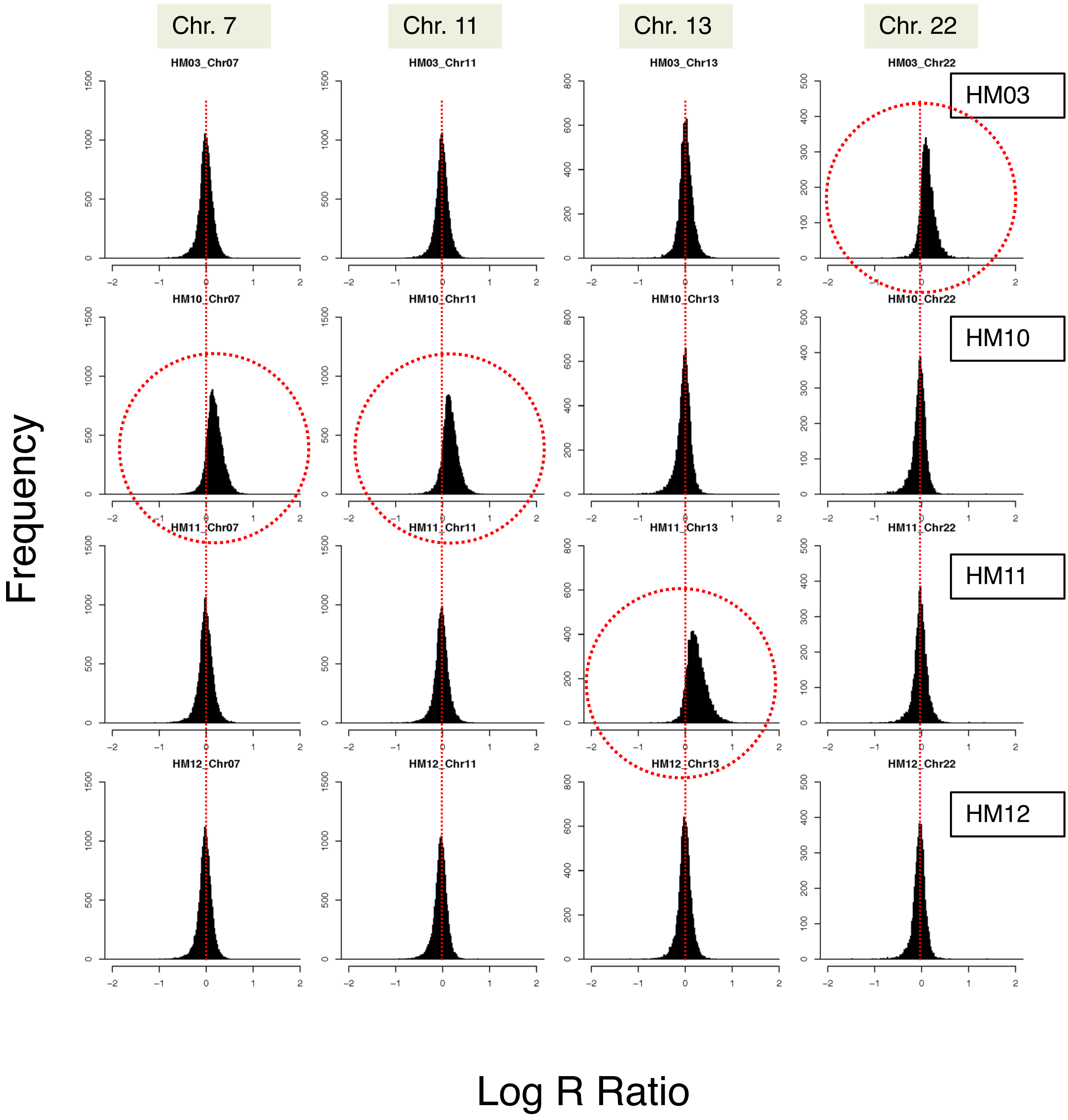

Chr. 7
Chr. 11
Chr. 13
Chr. 22
HM03
HM10
HM11
HM12
HM03_Chr07
HM03_Chr11
HM03_Chr13
HM03_Chr22
HM10_Chr07
HM10_Chr11
HM10_Chr13
HM10_Chr22
HM11_Chr07
HM11_Chr11
HM11_Chr13
HM11_Chr22
HM12_Chr07
HM12_Chr11
HM12_Chr13
HM12_Chr22
Frequency
Log R Ratio

Supplementary Figure S4:

Histograms of distribution of log R ratio on sex chromosomes. The value “0” of log R ratio on chromosome X indicates XX. The value “0” of log R ratio on chromosome Y indicates Y, but not YY. The histograms were prepared using the R software (https://www.R-project.org/).

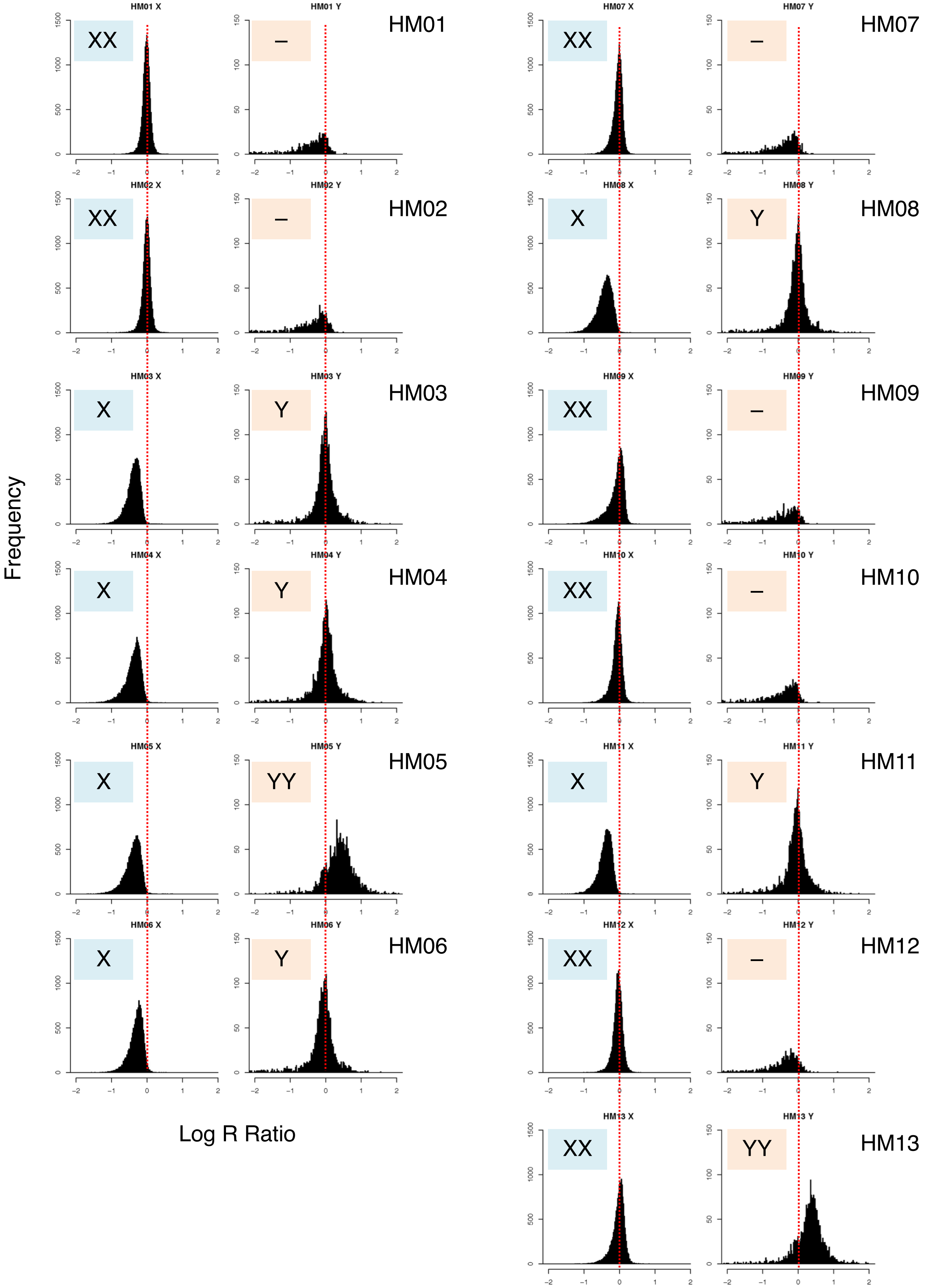

Frequency
Log R Ratio
HM01 X
XX
HM01 Y
–
HM01
HM02 X
XX
HM02 Y
–
HM02
HM03 X
X
HM03 Y
Y
HM03
HM04 X
X
HM04 Y
Y
HM04
HM05 X
X
HM05 Y
YY
HM05
HM06 X
X
HM06 Y
Y
HM06
HM07 X
XX
HM07 Y
–
HM07
HM08 X
X
HM08 Y
Y
HM08
HM09 X
XX
HM09 Y
–
HM09
HM10 X
XX
HM10 Y
–
HM10
HM11 X
X
HM11 Y
Y
HM11
HM12 X
XX
HM12 Y
–
HM12
HM13 X
XX
HM13 Y
YY
HM13

Supplementary Figure S5:

B allele frequency (BAF) plots and log R ratio (LRR) of sex chromosomes
(a) BAF and LRR plotting of HM03 (47,XY,+22) depicted under default condition (using “X”, “Y”, and “XY” probes). (b) BAF and LRR of HM03 depicted under filtered condition without “XY” probes (using “X” and “Y” probes). (c) BAF and LRR plotting of HM03 depicted under filtered condition only with “XY” probes. (d) Default condition of HM01(46,XX). (e) Default condition of HM05 (47,XYY). (f) Default condition of HM13 (48,XXYY). Grey filled squares are the regions without SNP probes around centromeres. Red dotted squares indicate pseudoautosomal region (PAR). Black dotted circles present the homologous region to the Y chromosome. Arrows indicates the regions that copy number was estimated to be two. Red arrow regions are assigned to “XY” probes, while black arrow region is not assigned to “XY” probes. Black circles indicate BAF plotting on PAR1 with sex chromosome polysomy.
BAF, B allele frequency; LRR, log R ratio.

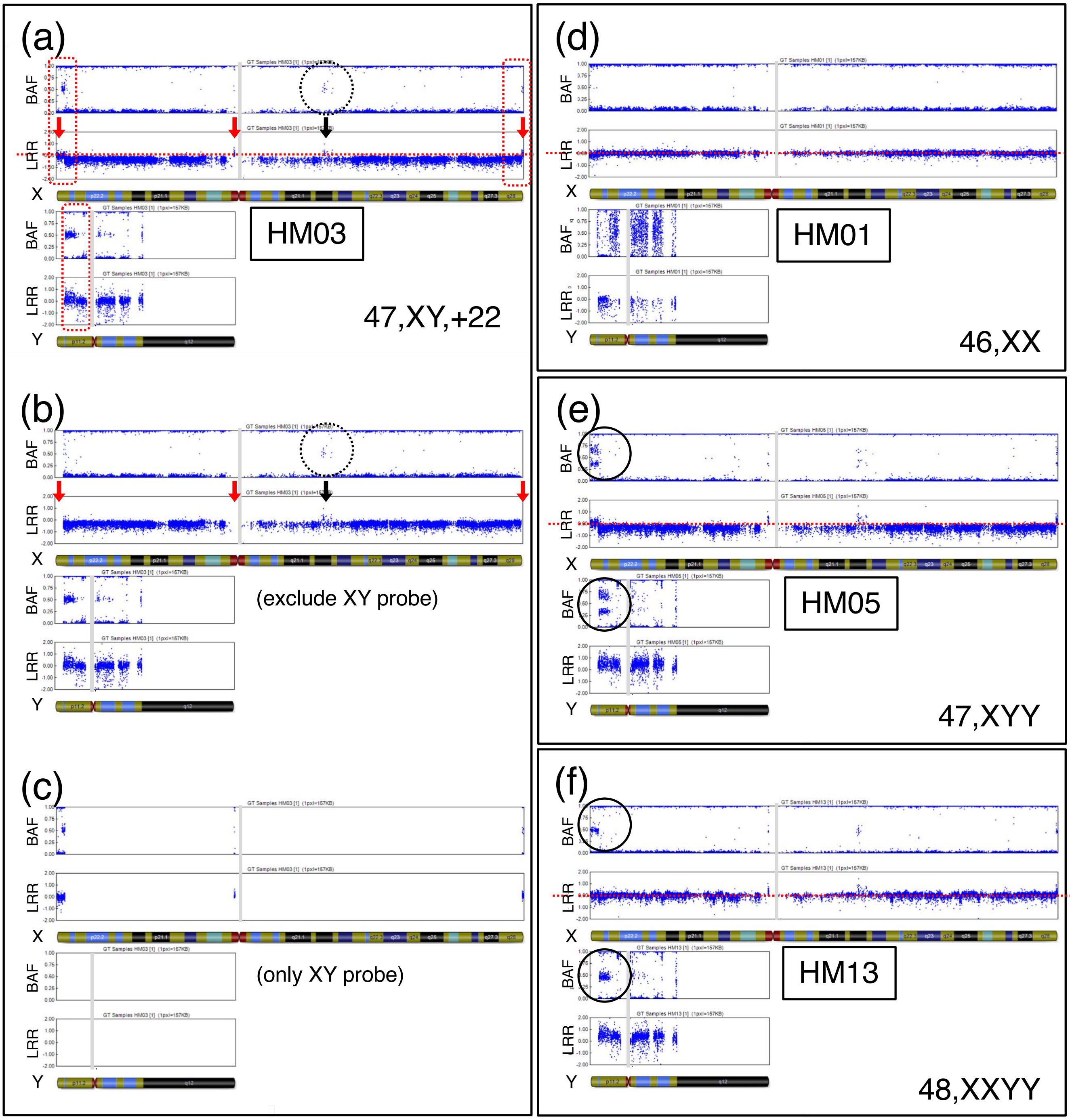
(a)
HM03
47,XY,+22
(b)
(exclude XY probe)
(c)
(only XY probe)
(d)
HM01
46,XX
(e)
HM05
47,XYY
(f)
HM13
48,XXYY
BAF
LRR
X
Y
